# Mining microsatellite markers from public expressed sequence tags databases for the study of threatened plants

**DOI:** 10.1186/s12864-015-2031-1

**Published:** 2015-10-13

**Authors:** Lua Lopez, Rodolfo Barreiro, Markus Fischer, Marcus A. Koch

**Affiliations:** Center for Organismal Studies (COS) Heidelberg/Botanic Garden and Herbarium Heidelberg (HEID), University of Heidelberg, Im Neuenheimer Feld 345, D-69120 Heidelberg, Germany; BioCost research group, Departamento de bioloxía animal, vexetal e ecoloxía, Facultade de Ciencias, University of A Coruña, E-15008 A Coruña, Spain; Institute of Plant Sciences, University of Bern, Altenbergrain 21, CH-3013 Bern, Switzerland

**Keywords:** Conservation, Evolution, EST-SSR, Functional markers, Population genetics, Threatened plants

## Abstract

**Background:**

Simple Sequence Repeats (SSRs) are widely used in population genetic studies but their classical development is costly and time-consuming. The ever-increasing available DNA datasets generated by high-throughput techniques offer an inexpensive alternative for SSRs discovery. Expressed Sequence Tags (ESTs) have been widely used as SSR source for plants of economic relevance but their application to non-model species is still modest.

**Methods:**

Here, we explored the use of publicly available ESTs (GenBank at the National Center for Biotechnology Information-NCBI) for SSRs development in non-model plants, focusing on genera listed by the International Union for the Conservation of Nature (IUCN). We also search two model genera with fully annotated genomes for EST-SSRs, *Arabidopsis* and *Oryza*, and used them as controls for genome distribution analyses. Overall, we downloaded 16 031 555 sequences for 258 plant genera which were mined for SSRsand their primers with the help of QDD1. Genome distribution analyses in *Oryza* and *Arabidopsis* were done by blasting the sequences with SSR against the *Oryza sativa* and *Arabidopsis thaliana* reference genomes implemented in the Basal Local Alignment Tool (BLAST) of the NCBI website. Finally, we performed an empirical test to determine the performance of our EST-SSRs in a few individuals from four species of two eudicot genera, *Trifolium* and *Centaurea.*

**Results:**

We explored a total of 14 498 726 EST sequences from the dbEST database (NCBI) in 257 plant genera from the IUCN Red List. We identify a very large number (17 102) of ready-to-test EST-SSRs in most plant genera (193) at no cost. Overall, dinucleotide and trinucleotide repeats were the prevalent types but the abundance of the various types of repeat differed between taxonomic groups. Control genomes revealed that trinucleotide repeats were mostly located in coding regions while dinucleotide repeats were largely associated with untranslated regions. Our results from the empirical test revealed considerable amplification success and transferability between congenerics.

**Conclusions:**

The present work represents the first large-scale study developing SSRs by utilizing publicly accessible EST databases in threatened plants. Here we provide a very large number of ready-to-test EST-SSR (17 102) for 193 genera. The cross-species transferability suggests that the number of possible target species would be large. Since trinucleotide repeats are abundant and mainly linked to exons they might be useful in evolutionary and conservation studies. Altogether, our study highly supports the use of EST databases as an extremely affordable and fast alternative for SSR developing in threatened plants.

**Electronic supplementary material:**

The online version of this article (doi:10.1186/s12864-015-2031-1) contains supplementary material, which is available to authorized users.

## Background

Since the 1980s, the fast advent of molecular markers technology has revolutionized the field of genetics by changing the pace and accuracy of genetic analysis. Today, the analysis of DNA variation is a key component in plant genetics studies addressing relevant aspects such as evolution, phylogeny or conservation [1–3]. Among the various types of molecular markers used for these purposes, Simple Sequence Repeats (SSRs) are often regarded as the markers of choice because of their abundance, multiallelic behavior, high polymorphism and codominant inheritance [4]. Despite that the recent development of next generation sequencing (NGS) techniques has facilitated the *de novo* development of SSRs, this task is still quite expensive and requires a substantial amount of time [5]. Furthermore, genomic SSRs are usually species-specific, meaning that markers developed for one taxon are not always directly transferred to another [6]. In fact, the rates of successful cross-species transferability vary greatly between taxonomic groups [7].

With the recent and growing emphasis on functional genomics, the number of large datasets of DNA sequences generated by high-throughput technologies has largely increased for a wide variety of taxa. In this context, Expressed Sequence Tags (ESTs) databases available for public use arise as an attractive alternative for SSR mining and development [8–10]. Microsatellites generated from ESTs (i.e. EST-SSRs) display several advantages over those derived from anonymous DNA regions. First, time and costs for SSR development are considerably lower. Instead of the weeks required for SSRs development with conventional approaches, it takes 2–3 days to obtain a batch of EST-SSR markers, together with the primers needed for their testing, from existing databases. Second, any type of SSR motif can be detected in EST-SSR mining while a subset of predefined motifs are favored in conventional approaches which involve an enrichment cloning step. Third, SSRs have been found to be moderately abundant (≈2-5 %) also in EST sequences due to the preferential association with the non-repetitive fraction of the plant genome [11, 12]. Finally, EST-SSRs located in conserved regions are highly transferable between related species, and often even genera, because the conserved flanking sequences are ideally suited for primer design [13, 14]. The latter facilitates comparisons among related taxa for addressing the mechanisms behind population diverge and speciation as well as comparisons among several co-occurring species [15, 16].

Nevertheless, EST-SSRs also pose some challenges. Their development is restricted to organisms with existing EST databases; although SSR mining from EST sequences of related species is also a promising alternative. In addition, EST-SSRs are expected to display lower levels of polymorphism than anonymous SSRs as they are associated with conserved regions of the genome [9, 17]. Nonetheless, several studies with EST-SSRs found moderate to high level of polymorphism [18–20]. Finally, another possible concern regarding EST-SSRs is that these often non-neutral loci might bias the estimates of population divergence under the assumption of a neutral model of drift, mutation and migration [21]. However, several studies reported that population structure measures derived from EST-SSRs were consistent with those from anonymous SSRs, and as a matter of fact, only a very small fraction of all genes might have experienced recent positive selection [22–24].

EST-SSRs can be considered “functional markers” because ESTs represent a portion of the transcribed region of the genome under certain conditions [17, 25]. For a majority of these markers, a “putative function” can be deduced by comparison against annotated reference genomes. Dinucleotide repeats in ESTs are known to be favored in Untranslated Regions (UTRs) and introns, while trinucleotide repeats are frequently associated to coding regions [12]. Thus, compared with anonymous microsatellites, EST-SSRs offer the opportunity to detect variation in the transcribed portion of the genome that could show a marker-trait association [17].

To date, EST-SSR markers have been successfully used for resolving phylogenies [26] and to increase resolution in comparative genetic mapping studies by cross-referencing genes between species [13, 27]. These studies have been mainly focused on species with economic relevance and model species [11, 28–31]. Even if EST-SSRs can be regarded as a potential tool for addressing evolutionary and conservation-related questions in threatened plant species, their application in these type of studies has been overlooked and examples in the literature are limited [20, 32].

The present study explores the development of markers from public EST databases for evolutionary and conservation genetic studies of non-model plants, with special emphasis in threatened species. We searched all plant genera included in the International Union for the Conservation of Nature (IUCN) Plant Red List which had EST sequences available in the dbEST database (GenBank at the National Center for Biotechnology Information-NCBI). Since most of these plant genera do not include model organisms, normally there are no available annotated reference genomes for comparison, hampering the location of the EST-SSRs within the genome. Since the location of the EST-SSRs across the different regions in the genome might have implications in the analysis and interpretation of the results, we analyzed in depth the EST sequences data sets for two model genera with well-known annotated genomes and used them as a proxy. By doing so, we aimed to identify general distribution patterns of the various types of repeats and motifs along the different regions of the genome that can be applied for the remaining analyzed genera. The genus *Arabidopsis* was selected as a control for Eudicotyledoneae while *Oryza* was used as a guide for Monocotyledoneae. Finally, a proof-of-concept study was undertaken by testing for amplification, cross-amplification and polymorphism of 24 EST-SSRs in four species from two genera (*Trifolium fragiferum* L.*, Trifolium saxatile* All.*, Centaurea valesiaca* (DC.) Jord and *Centaurea borjae* Valdés-Bermejo & Rivas Goday). These four species are of conservation interest due to their threatened status: *T. saxatile* is listed as near threatened [33] and *C. borjae* as endangered [34] by the IUCN, while *T. fragiferum* is catalogued as vulnerable and *C. valesiaca* as near threatened in the Swiss Red List of endangered vascular plants [35].

## Methods

### Sequence data sources

By September 2013, 16 031 555 sequences were downloaded from the dbEST database in GenBank at the NCBI website (http://www.ncbi.nlm.nih.gov/dbEST/). Batch files of EST sequences were downloaded in FASTA format. The dataset included 14 498 726 records for 257 genera (*Oryza* included) listed both in IUCN Red List and dbEST plus 1 532 829 records for *Arabidopsis*.

### EST-SSRs detection and primer design

SSRs were detected in the EST datasets with the help of QDD1 [36]. Before SSR search, QDD1 assembled the ESTs of each genus into unique sequences (contigs and singletons) to avoid redundancy. Non-redundant EST sequences were then screened for perfect SSRs. In the present study only Class I microsatellites, defined as DNA sequences containing at least 20 bp, were considered [37]. That is ten repeats for di-, seven for tri-, five for tetra- and four for penta- and hexanucleotide repeats respectively. Mononucleotide repeats were excluded from the EST-SSRs search as their polymorphism is often difficult to interpret. To have enough flanking sequence of appropriate quality for primer design, only EST sequences larger than 100 bp were taken into account during EST-SSR searches. EST-SSR primers were designed with the version of Primer3 embedded in QDD1 [38] under the following criteria: length of primers ranging from 18–23 nucleotides (optimum 20 bp), annealing temperature 55–65 °C (optimum 60 °C), GC content 30–70 % (optimum 50 %) and PCR product size from 90 to 320 bp.

### Basal local alignment search tool (BLAST) searches in *Oryza* and *Arabidopsis*

EST sequences for the control genera, *Oryza* and *Arabidopsis*, were run in QDD1 following the criteria specified above. QDD1 output files were then used as inputs for the BLASTn search against *Oryza sativa* and *Arabidopsis thaliana* reference genomes using default parameters specified on the NCBI website. Whenever a positive hit was found, the matching gene sequence was downloaded and aligned in Geneious 6.1.6 (created by Biomatters, available from http://www.geneious.com/). The distribution of the SSRs towards the genome (i.e. UTRs, exons, introns, genomic regions) was inferred using the annotated gene information derived from the BLASTn search. As double-check, a BLASTx search against the *Oryza* and *Arabidopsis* reference protein databases was also conducted for EST-SSRs using the megablast option with the default algorithm parameters.

### Compositional analysis of SSR mining

Occurrence and frequency of SSR motifs in the IUCN genera were analyzed after importing QDD1 output files into MATLAB and Statistics Toolbox 2013a (MathWorks Inc., MA, US) (Additional file 1). Repeat types, number of repeats, and frequency were calculated for each genus using a combination of sorting and counting functions. Results were displayed using tabular and graphical representations. To provide a broader view, results from the IUCN genera were grouped in eight taxonomic groups: Florideophyceae, Charophyceae, Monilophyta, Lycopodiophyta, Acrogymnospermae, Magnoliidae, Monocotyledoneae and Eudicotyledoneae [39].

### DNA isolation, PCR conditions, and amplification of SSRs

Six individuals of *Trifolium fragiferum,* seven from *Centaurea valesiaca,* two individuals of *Trifolium saxatile* and two from *Centaurea borjae* were used for the screening of EST-SSR amplification. Fresh leaves were dried in silica gel until DNA extraction. Leaf tissue from each plant was collected in a 2.0 ml Eppendorf tube, frozen with liquid nitrogen and ground to fine powder with a Mini-BeadBeater (Glen Mills Inc, NJ, US). DNA was extracted using the Wizard Magnetic Kit (Promega, Madison, WI, US) according to manufacturer instructions. The quality of the extracted DNA and negative controls were checked in 1.5 % agarose gels. Twelve primer pairs were selected for each genus to test the EST-SSRs amplification. Amplification was tested with a standard PCR reaction performed in 25 μl containing 1x reaction buffer (NzyTech, Lisboa, Portugal), 2 mM MgCl_2_ (NzyTech), 0.2 μM of each dNTP (Fermentas GmBH, St. Leon-Rot, Germany), 0.16 μM of each primer, 1 μl of genomic DNA and 0.5 units of DNA polymerase (NzyTech). PCR profiles consisted of 5 min denaturation at 94 °C followed by 35 cycles of 30 s denaturation at 94 °C, 50 s annealing at 59 °C, 45 s of extension at 72 °C, with a final elongation step of 35 min at 72 °C. PCR products were screened on 2 % agarose gels. Primer pairs that had successfully amplified in the first round where re-tested with the M13 tail method [40]. PCR reactions were performed following the procedure specified above with the addition of 0.04 μM of the forward primer with the M13 tail and 0.16 μM of the reverse and 0.16 μM of the M13-FAM primer. PCR profiles comprised 5 min denaturation at 94 °C followed by 35 cycles of 30 s denaturation at 94 °C, 50 s annealing at 59 °C, and 45 s of extension at 72 °C, followed by eight additional cycles of 30 s denaturation at 94 °C, 45 s annealing at 53 °C, 45 s of extension at 72 °C, and a final elongation step of 35 min at 72 °C. PCR products were screened on 2 % agarose gels and sized on an ABI-3730XL DNA analyzer (Applied Biosystems, Foster City, CA, US) using a 500HD size ladder. PCR reactions from one primer pair that produced PCR amplicons larger than expected were purified with 1 μl of Exonuclease I (20 u/μl) (Fermentas GmBH) and 2 μl of FastAP (10 u/μl) (Fermentas GmBH and bi-directionally sequenced (BigDye Terminator cycling conditions) in an Automatic Sequencer 3730XL (Applied Biosystems).

## Results

### Frequency and distribution of SSRs in *Arabidopsis* and *Oryza*

**Fig. 1:**
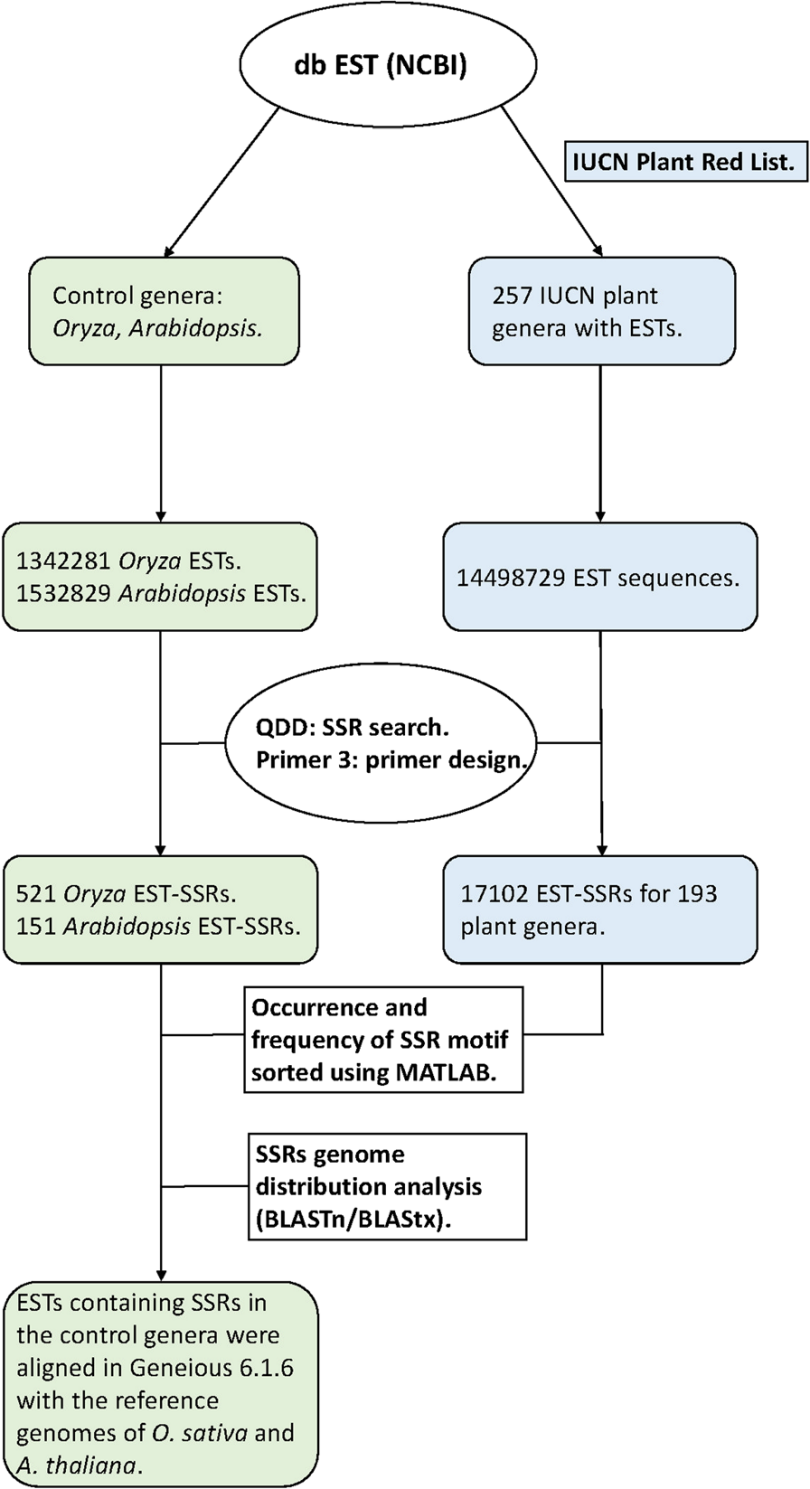
Flowchart of bioinformatics analysis used for developing the EST-SSR. The results of the SSR mining in the control genera *Oryza* and *Arabidopsis* are indicated in green on the left side of the figure while in blue, on the right side, are shown the results of the SSR mining in the IUCN plant genera (note that *Oryza *was used as a control genus and also included in the IUCN analyses). The steps followed for the analysis are highlighted with bold letters in the center

The dbEST database contained 1 342 281 *Oryza* EST sequences (Fig. 1). After filtering redundant sequences and those shorter than 100 bp, 2 626 EST sequences (1 912 singletons and 714 contigs) remained for the microsatellite and primer search. From those, QDD1 found 521 perfect EST-SSRs with primer pairs (19.2 %). On the other hand, the *Arabidopsis* dataset encompassed 1 532 829 EST sequences that, after filtering, was reduced to 899 EST sequences (616 singletons and 283 contigs) that contained 151 perfect microsatellites with primer pairs (16.8 %) (Fig. 1). In both cases, filtering had a large impact on the number of EST records available for SSR search, indicating a high rate of redundant and/or short records in the EST database.

Although only sequences assigned to *Oryza* were downloaded from the dbEST, 23.8 % of the analyzed EST sequences containing SSRs did not render a significant hit in the BLASTn search against the *Oryza sativa* reference genome. Similarly, the BLASTn comparison of *Arabidopsis* EST sequences containing SSRs against the *Arabidopsis thaliana* reference genome had 8 % of unsuccessful searches. The microsatellites derived from these sequences were excluded from further analysis. Thus, the distribution and position of 397 and 139 EST-SSRs were determined for *Oryza* and *Arabidopsis*, respectively (Table 1). Trinucleotide repeats were the commonest repeat size in both genera with very similar relative abundances (61.96 % in *Oryza* and 69.78 % in *Arabidopsis*). Dinucleotide repeats were second in abundance (23.29 % in *Oryza* and 17.27 % in *Arabidopsis*), while tetra- and pentanucleotide repeats were scarce in both genera (<5 %). Hexanucleotide repeats displayed intermediate frequencies in both genera (11.59 % in *Oryza* and 8.63 % in *Arabidopsis*).

**Table 1:** Number and distribution of the EST-SSRs found for the EST sequences of *Oryza* and *Arabidopsis*

	Genomic	Intron	UTR	Exon	Total					
	*Oryza*	*Arabidopsis*	*Oryza*	*Arabidopsis*	*Oryza*	*Arabidopsis*	*Oryza*	*Arabidopsis*	*Oryza*	*Arabidopsis*
Dinucleotides	17	2	26	5	29	16	1	1	73	24
Trinucleotides	18	2	16	2	70	26	142	67	246	97
Tetranucleotides	3	0	3	0	9	3	2	0	17	3
Pentanucleotides	4	0	1	0	10	3	0	0	15	3
Hexanucleotides	6	1	3	0	13	1	24	10	46	12
Total	48	5	49	7	131	49	169	78	397	139

SSR search was only carried out in those EST sequences downloaded from the dbEST database (NCBI) that had a match in their respective reference genomes using BLASTn

**Fig. 2:**
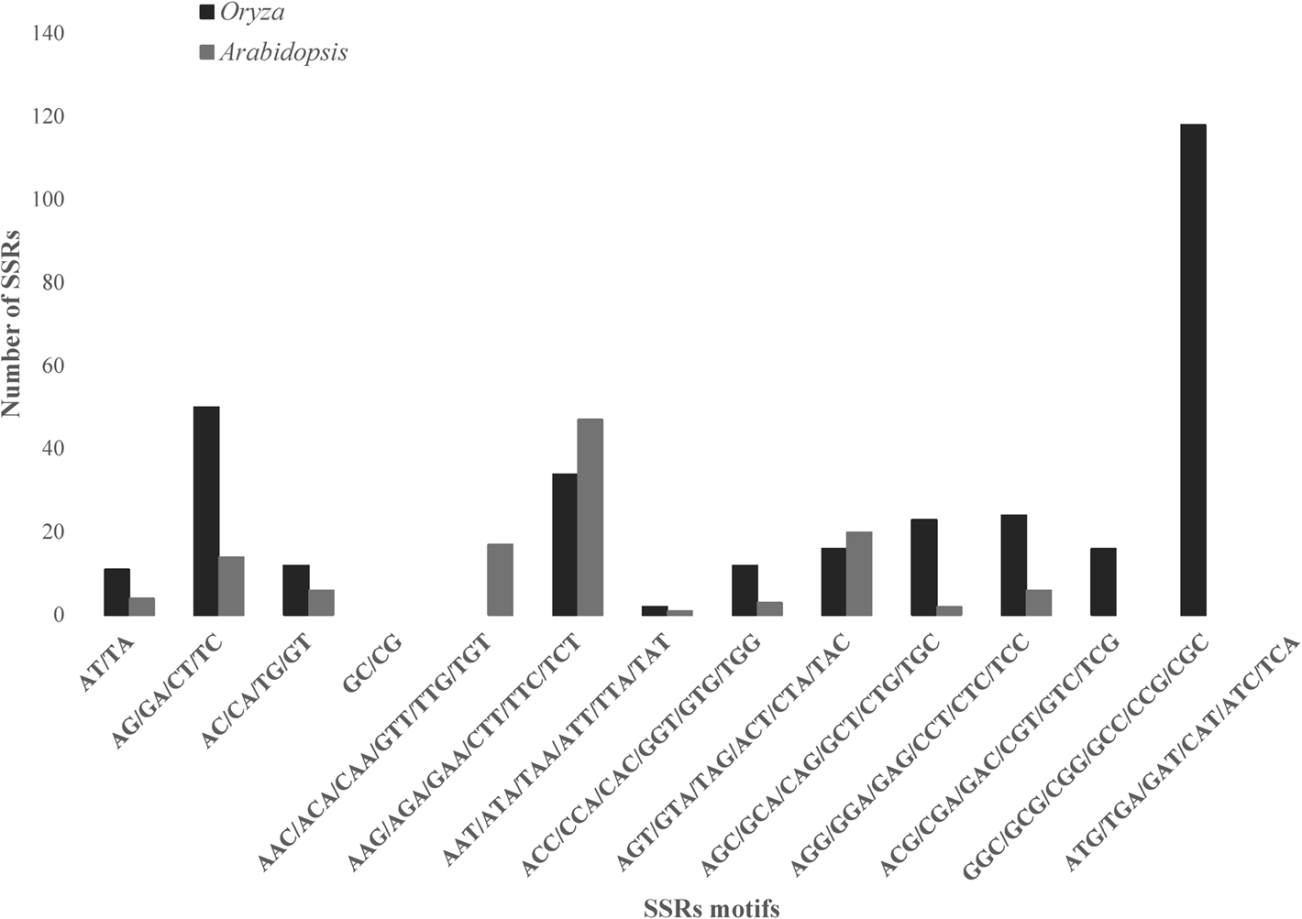
Di- and trinucleotides distribution obtained using QDD1 software from *Oryza* and *Arabidopsis* EST sequences that had a positive hit in the *Oryza sativa* (japonica cultivar-group) and *Arabidopsis thaliana* reference genomes database with BLASTn (NCBI). *Oryza* is represented in black while *Arabidopsis* is displayed in grey. The different types of motif are detailed in axis X while the number of SSRs for each class are showed in axis Y

The various SSR motifs were grouped into classes according to base complementarity and depending on the reading frame (for groups see Fig. 2; from now on, in the text they will be identified with the first motif repeat). Motifs in dinucleotide repeats displayed similar patterns in both genera as the AG group was the most abundant while the AC and AT groups were scarce, and those from the GC group went undetected (Fig. 2)*.* Despite that the AG group prevailed in both genera, it was clearly commoner in *Oryza* than in *Arabidopsis*. Unlike dinucleotide, trinucleotide repeats displayed different patterns in each genus. Various motifs from trinucleotide repeats which were found in *Oryza*, went unrecorded (GGC and ACG) or very rare (AGC, ACC and AGG) in *Arabidopsis*. Likewise, the AAC group found in *Arabidopsis* was absent in *Oryza.* The group GGC dominated in *Oryza,* while motifs from the AAG, AGC and AGG groups had intermediate values, and the AAT group was clearly underrepresented (Fig. 2). In comparison, trinucleotide repeats in *Arabidopsis* were dominated by the AAG group, while AGC and AAT groups were very scarce (i.e. only one and two SSR detected, respectively). No motif of the ATG group was found for either genera.

Using the alignments of the EST sequences containing SSRs and the reference genomes downloaded from GenBank, four possible location categories were established based on to the information provided by the BLASTn results: introns, untranslated regions (UTRs), exons and genomic region (when the EST-SSR did not fall in any of the other categories). Overall, the majority of EST-SSRs were located in exons (42.57 % in *Oryza* and 56.12 % in *Arabidopsis*) followed by UTRs (33.00 % in *Oryza* and 35.25 % in *Arabidopsis*) and only a small fraction of the EST-SSRs was found in introns (12.34 % in *Oryza* and 5.04 % in *Arabidopsis*) and genomic regions (12.09 % in *Oryza* and 3.60 % in *Arabidopsis*) (Table 1). The proportion of EST-SSRs found in introns and genomic regions in *Oryza* was more than two and four times larger than in *Arabidopsis*, as it would be expected due to the compact and small genome of *Arabidopsis*. Repeats of different size showed characteristic locations along the genome. Thus, tri- and hexanucleotide repeats were mostly concentrated in exons in both genera (57.72 % and 69.07 % of the total number of trinucleotide repeats in *Oryza* and *Arabidopsis*, and 52.17 % and 83.33 % of the total number of hexanucleotide repeats in *Oryza* and *Arabidopsis*). In contrast, dinucleotide repeats mostly occurred in non-coding regions, mainly in UTRs (39.73 % of the total dinucleotide repeats in *Oryza* and 66.67 % in *Arabidopsis*) but also in introns (35.62 % of the total dinucleotide repeats in *Oryza* and 20.83 % in *Arabidopsis*) and genomic regions (23.29 % of the total dinucleotide repeats in *Oryza* and 8.33 % in *Arabidopsis*). Tetra- and pentanucleotide repeats were scarce and they occurred almost only associated to non-coding regions (except for 11.76 % of *Oryza*’s pentanucleotide repeats which were located in exons).

### EST-SSRs analysis from the IUCN genera

Two hundred and fifty-seven genera included in the IUCN plant red list were mined for SSR using the EST sequences available in the dbEST (NCBI) (Fig. 1). These genera included two Florideophyceae, one Charophyceae, three Lycopodiophyta, five Monilophyta, five Magnoliidae, 18 Acrogymnospermae, 58 Monocotyledoneae and 165 Eudicotyledoneae. Overall, 14 498 726 sequences were screened for SSR discovery (Table 2). In a few cases, SSR search and primer design were unsuccessful due to a very low number of EST sequences in the input file or sequences that did not fulfill the predefined criteria. As a result, 193 genera were successfully mined for SSRs rendering 17 102 microsatellites with their respective primers (see Additional file 2: Table S1). From the total number of EST-SSR, the largest proportion belonged to Eudicotyledoneae covering 73.19 %, followed by Monocotyledoneae (18.17 %) and Acrogymnospermae (8.29 %) while each of the remaining groups had <1 % frequency. The percentage of SSR found was related with the number of EST sequences downloaded, for example, the group Eudicotyledoneae represented 67.19 % of the total number of EST sequences and the Monocotyledoneae 22.05 %. Nevertheless, the latter is not true for the Acrogymnospermae where the number of EST sequences analyzed were 8.20 % and the frequency of EST-SSR was 3.33 %.

**Table 2:** Number of ETS-SSRs found in the IUCN plant genera containing EST sequences in the dbEST

Taxonomic groups	Ng	Ng _SSR_	N_EST_	Dinucleotides	Trinucleotides	Tetranucleotides	Pentanucleotides	Hexanucleotides	Total	Commonest motifs
Florideophyceae	2	2	16645	2	10	2	1	10	25	ACG/GGC
Charophyceae	1	1	88280	16	77	39	38	30	200	AG/TGA
Acrogymnospermae	18	15	1191184	144	145	30	58	193	570	AG/AT/CAG
Lycopodiophyta	3	3	101292	20	122	15	7	26	190	AG/CAG/TGA
Monilophyta	5	3	35665	129	18	3	2	6	158	AG/TGA
Magnoliidae	5	5	68569	193	89	11	9	30	332	AG/AT/CAG
Monocotyledoneae	58	37	3197142	598	1395	296	323	496	3108	AG/AT/AAG/CGG
Eudicotyledoneae	165	127	9742277	4160	4820	760	769	2010	12519	AG/AT/AAG/TGA
Total	257	193	14498726	5262	6676	1156	1207	2801	17102	

*Ng* number of genera, *Ng*_*SSR*_ *, N*_*EST*_ number of EST sequences downloaded, number of genera with SSRs

**Fig. 3:**
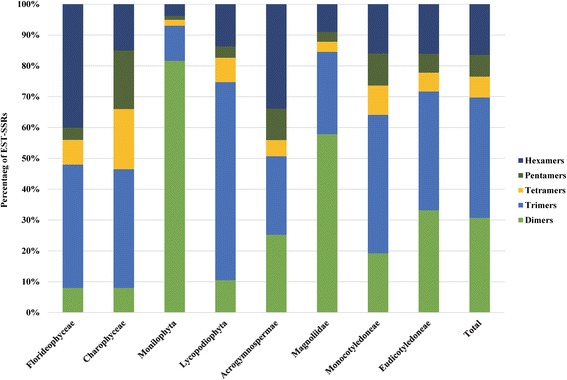
Distribution of EST-SSRs in 193 plant genera including threatened species by the IUCN. Bars in the X axis represent each taxonomic group investigated and the whole dataset. The axis Y represents the percentage of EST-SSRs found within each group. Colors in each bar indicate the type of repeat: dinucleotide repeats in light green, trinucleotide repeats in light blue, tetranucleotide repeats in yellow, pentanucleotide repeats in dark green and hexanucleotide repeats in dark blue

As in the control genomes, di- and trinucleotide repeats were the commonest types of SSR (30.76 % and 39.03 % respectively) while tetra- and pentanucleotide repeats were very scarce (6.76 % and 7.06 % respectively), and hexanucleotide repeats displayed an intermediate position (16.38 %). Nonetheless, when the frequency of the various classes of SSR was analyzed in detail, there were differences among taxonomic groups (Fig. 3). Trinucleotide repeats were commoner than dinucleotide repeats in eudicots (38.50 % vs. 33.23 %) and monocots (44.88 % vs. 19.24 %). In Acrogymnospermae, hexanucleotide repeats dominated representing more than one third of the SSRs followed by di- and trinucleotide repeats with a 25 % frequency each. Furthermore, trinucleotide repeats prevailed in Lycopodiophyta (64.21 %), while dinucleotide repeats dominated in Monilophyta (81.65 %) and Magnoliidae (58.13 %). In Florideophyceae tri- and hexanucleotide repeats displayed the highest frequencies (40 % each type of repeat). Finally, tetra- and pentanucleotide repeats were rare across all groups (≤10 % each type) except in Charophyceae where each one represented almost 20 % of the total.

Overall, the most abundant dinucleotide repeats were from the AG group. For trinucleotide repeats there was no consensus along all the groups studied, but overall the AGT and AGC groups were the commonest. When each taxonomic group was considered separately, the AT group was very common in Acrogymnospermae while in red algae the ACG and GGC groups were the most frequent. Moreover, the GC-rich trinucleotide repeats displayed high abundance in Monocotyledoneae while they were absent from the remaining groups. Tetra-, penta- and hexanucleotide repeats were too scarce in most taxa to allow an appropriate analysis of their distribution.

### Amplification and transferability of the EST-SSRs

A subset of 24 pairs of EST-SSR primers (12 pairs per genus) were chosen to test amplification performance in two genera of Eudicotyledoneae, *Trifolium* and *Centaurea* (Table 3). A total of 53 422 *Trifolium* EST sequences were run for SSR search. The ESTs data set of *Trifolium* included three species *Trifolium pretense* L., *Trifolium repens* L. and *Trifolium purpureum* Loisel, with 38 109, 15 260 and 53 EST sequences respectively. The SSR search rendered 130 EST-SSRs with their primers; 23 were di-, 77 tri-, 11 tetra-, 9 penta- and 10 hexanucleotide repeats (Additional file 2: Table S1). From those, 12 EST-SSRs were selected; three di-, seven tri- and two tetranucleotide repeats. Six of the selected EST-SSRs derived from unique sequences of *T. repens* and four from unique sequences of *T. pratense*, while the two remaining derived from contigs of *T. pratense* (Table 3). Likewise, the 85 293 EST sequences analyzed for *Centaurea* comprehended two *Centaurea* species, *Centaurea maculosa* Lam. and *Centaurea sosltitialiis* L. with 44 886 and 40 407 EST sequences respectively. The *Centaurea* EST sequences returned 306 EST-SSRs with their primers; 24 were di-, 146 tri-, 33 tetra-, 26 penta- repeats and 77 hexanucleotide repeats. From those, 12 EST-SSRs were selected; three di-, six tri-, two tetra- and one hexanucleotide repeat. Five of the selected EST-SSRs derived from unique sequences of *C. solstitialis* and five from unique sequences of *C. maculosa*, while the two remaining derived from contigs of *C. maculosa* and *C. solstitialis* respectively (Table 3). Overall, thirteen out of the 24 pairs of primers yielded a clear amplification product (amplification rate 54.2 %). Nevertheless, the amplification success differed between genera and *Centaurea* displayed a higher amplification rate (eight out of 12 EST-SSRs, 58.3 %) than *Trifolium* (five out of 12 EST-SSRs, 41.7 %).

**Table 3:** EST-SSRs tested empirically in two Eudicotyledoneae genera, *Trifolium* and *Centaurea*

Locus	GenBank accession No.	Species	Primer sequences		Repeat motif	Expected size (bp)	*N*_A_	Size range (bp)
			Forward	Reverse				
T6-*Trifolium*	gi86106666	*T. pratense*	CAACCAGTGGTGTGAGTAGGAG	ACGTTGGTGGAGAGGTTGAG	(AG)_11_	110–128	2	114–116
	gi86105378							
T7-*Trifolium*	gi428283538	*T. repens*	ATCACGCTTCACTCCTCCAC	CAACTCCAAGCTTAAGATCGTGTA	(AG)_13_	110–122		no PCR product
T1-*Trifolium*	gi428292074	*T. repens*	AGATTCCCACCAATCTCCCT	CAATACGCGGGTCTTGATCT	(AG)_11_	210–228	---	257–261
T2-*Trifolium*	gi86106666	*T. pratense*	TTCCGGTTAGGTTAGGGTTT	TTTTCACATCTTCCGAAGCC	(AAT)_7_	110–113		no PCR product
	gi86105378							
T3-*Trifolium*	gi428285635	*T. repens*	CACCACATATGCAACCACAA	GTCGACGACGGTTGTTACCT	(AGT)_8_	110–126		no PCR product
T8-*Trifolium*	gi428291122	*T. repens*	GCAAAACTCAAGAGAACGGC	GGATGTCTTCGGAGGTGAGA	(ACC)_7_	110–122		no PCR product
T9-*Trifolium*	gi428292435	*T. repens*	ACAACCCATTTGCCTCAAAG	TTTTCACTTCCACCACCTCC	(ACC)_7_	110–133	2	124–127
T10-*Trifolium*	gi86119186	*T. pratense*	TCCACTAGTTCTAGAGCGGC	TCCTGTAAACTGGAGGAGCC	(ACC)_9_	110–153		no PCR product
T11-*Trifolium*	gi86124411	*T. pratense*	TGGCGGTGGTGACTTATACA	TGTTTGGCAGTGGTGATGTT	(AGG)_8_	110–153		no PCR product
T4-*Trifolium*	gi86125686	*T. pratense*	GCTGCCACAGCACTACCAG	AATATTACCGTGAATGAAGCTCAG	(ACC)_8_	110–113	1	110
T5-*Trifolium*	gi86097190	*T. pratense*	TGAGTTCCGAGTTAAGGCTCA	TTCGGTAACTCCGAGGATTG	(ACCT)_5_	210–217	2	227–230
T12-*Trifolium*	gi428282514	*T. repens*	GATTATTCAACCAAACGCCG	TAGAAAGCCACGCCAAGACT	(AATCC)_20_	290		no PCR product
C6-*Centaurea*	gi124618051	*C. maculosa*	TGGGATGCAGTCCAGTCATA	TTGCAACTTGCCTGTACCAC	(AC)_11_	160–162	1	256
C1-*Centaurea*	gi148298213	*C. maculosa*	GGGAACCACACCTTTCATCT	GATCTGGCTTGACCCAAGAA	(AC)_10_	90–119	2	99–101
C7-*Centaurea*	gi124669731 gi124688599	*C. solstitialis*	TCGTTTTCCGATCACAAACTC	CAATTTGGCGACATCTCCTT	(AC)_12_	110–160	4	114–152
C2-*Centaurea*	gi124680442	*C. solstitialis*	CGCATTATGGAATAAACCCG	GCTTTCGACTTCATAAGCGG	(AAG)_7_	140–152	1	147
C8-*Centaurea*	gi148296795	*C. maculosa*	CGATGTATACAGGTGGTGCG	GGAGAAGGGGAGACGTAAGG	(ACC)_7_	110–150	2	141–144
C9-*Centaurea*	gi124675484	*C. solstitialis*	AACGGTAGGAACCAGCATTG	GATCCTCTGGCAGGGTCATA	(ACC)_9_	260–302	4	290–299
C10-*Centaurea*	gi124661102	*C. solstitialis*	AGTTGCCAGAAAGGAGCAAG	TCGAGAACAATGGCCTATCC	(AGC)_7_	210–229		no PCR product
C11-*Centaurea*	gi148292432	*C. maculosa*	TCCATGGATACAACCACCAA	GCGATATTCGGATGCAAAGT	(AGG)_7_	160–175	4	160–172
C3-*Centaurea*	gi124632630	*C. maculosa*	GCCATCCCCTTCTCTACTCC	GTTACAGGTGACGATGGGG	(AGT)_7_	160–181		no PCR product
C4-*Centaurea*	gi124691992	*C. solstitialis*	CTGCACCTACCCAGAGAAGC	CGGGAGAGGGTAAATTGTGA	(AGGT)_5_	110–115	3	103–109
C12-*Centaurea*	gi124632477	*C. maculosa*	ATGCATTGAGAAGGCCAATC	AACTCGCAAGCCTTTTCAAG	(AATCGG)_4_	210–223		no PCR product
C5*-Centaurea*	gi124673348	*C. solstitialis*	TTAAGCATTCTTCGAGGCGT	TCTATGCCTACGCCGATCTC	(AAGCAG)_5_	110		no PCR product
	gi124676118							
	gi124669484							

GenBank accession No., identification number of the EST sequences (when more than one ID refers to consensus sequence); species, indicates the species of the EST sequences; primer sequences; type of repeated motif; expected size of the PCR product; *N*_A_, number of alleles for the examined individuals and size range of the PCR product (−− indicates stutter peak)

All loci produced amplification products of the expected size, except for locus C6 of *Centaurea* that generated an amplicon longer than expected, suggesting the presence of a non-transcribed intron inside; which was further confirmed by sequencing the PCR product. The M13-tail protocol had mostly no impact on PCR performance since all pairs of primers that amplified in the unmodified state (first round of amplification) were also functional with an M13-tail attached. However, locus C7 produced a larger unspecific second band with the M13-tail method.

Despite the small number of individuals used in the empirical test, three out of the seven EST-SSRs that yielded a PCR product of the expected size in *Centaurea* displayed polymorphism in the seven individuals of *Centaurea valesiaca* (loci C1, C7 and C11 produced two, two and three genotypes) while for the two individuals of *Centaurea borjae* loci C7, C9 and C11 were polymorphic. Four loci, C4, C7, C9 and C11, showed variability between *Centaurea* species (Table 4). On the other hand, one of the dinucleotide repeats of *Trifolium* (loci T1) displayed a stutter-peak profile and was discarded from further analysis. Among the four remaining loci T5 displayed polymorphism in *Trifolium fragiterum* while loci T5 and T9 were polymorphic in *Trifolium saxatile*. Two loci, T6 and T9, showed variability between species (Table 4).

**Table 4:** Cross-species transferability of EST-SSRs in two plant genera, *Trifolium* and *Centaurea*

Locus	*N*_A_	Size Range (bp)	*N*_A_	Size Range (bp)
	*Centaurea valesiaca* (*n* = 7)		*Centaurea borjae* (*n* = 2)	
C6-*Centaurea*	1	256	1	256
C1-*Centaurea*	2	99–101	2	90–101
C7-*Centaurea*	2	141–143	4	114–152
C2-*Centaurea*	1	305	1	305
C8-*Centaurea*	1	144	1	141
C9-*Centaurea*	1	290	3	293–299
C11-*Centaurea*	2	160–166	3	160–169
C4-*Centaurea*	1	103	2	105–109
	*Trifolium fragiterum* (*n* = 6)		*Trifolium saxatile* (*n* = 2)	
T6-*Trifolium*	1	114	1	116
T1-*Trifolium*	--	257–261	--	257–261
T9-*Trifolium*	1	124	2	124–127
T4-*Trifolium*	1	110	1	110
T5-*Trifolium*	2	227–230	2	227–230

*n* number of individuals tested, *N*_*A*_ number of alleles for the examined individuals and size range of the PCR product (−− indicates stutter peak)

The selected primers were also used to assess the cross-species transferability in *Centaurea* and *Trifolium* (Table 4)*.* Cross-species transferability is considered successful when the EST-SSR is functional (i.e. one or two PCR product are present) and it is polymorphic (i.e. two or more alleles in the genera) [7]. Almost all the loci fulfilled the aforementioned criteria (75 %). All EST-SSRs that worked in one species were functional in its counterpart but three loci, C6 and C2 in *Centaurea* and loci T4 in *Trifolium*, were not polymorphic for the genera as only one allele was detected (Table 3).

## Discussion

Computational approaches allow the fast discovery of molecular markers from the ever-increasing publicly available genomic resources. Thus, SSRs derived from EST sequences arise as an excellent alternative to the classical techniques based on anonymous microsatellites because of their fast and inexpensive discovery [9]. Besides, unlike anonymous SSRs, EST-SSRs markers have been proven of great value in cross-species studies, linkage maps and discovering markers linked to genes [13]. So far, EST-SSRs have mainly targeted crop and model species [11, 29–31]. In contrast, the use of EST-SSRs in evolutionary and conservation studies with non-model species are still scarce [20, 32]. In this context, the present study has tried to fill this gap by providing EST-SSRs for plant genera listed by the IUCN which can be applied immediately in evolutionary and conservation genetic studies in a very large number of threatened species.

### Frequency and distribution of SSRs in *Arabidopsis* and *Oryza*

The frequency and distribution of microsatellites in EST sequences is highly variable among studies, in part because the efficiency of SSR discovery relies on several factors such as the mining tool used, the mining criteria, or the size of the EST sequences dataset [29, 41]. Differences in the mining criteria usually lead to significant deviations in the number of microsatellites identified in a given species using the same dataset [29]. Here, we opted for a conservative criteria and only Type I microsatellites were considered in an effort to increase the polymorphism of the detected ETS-SSRs [41]. As a consequence, we probably obtained a lower number of EST-SSRs than would have been found if more relaxed parameters had been set for the searching.

The in-depth analysis of the EST-SSRs frequency and their distribution in *Arabidopsis* and *Oryza* revealed that tri- and dinucleotide repeats encompassed more than 85 % of the total SSRs found. Furthermore, trinucleotide repeats comprehended the vast majority of the SSRs. High frequencies of trinucleotide repeats are known to be favored in higher plants and have been invariably reported in most studies [11, 24]. As expected in vascular plants, the AG group were the most abundant dinucleotide repeat motif and low frequencies of the group AT were recorded in both genera [11, 12, 24, 37]. In agreement with previous studies of monocots and dicots, we found differences in the trinucleotide repeats of *Oryza* and *Arabidopsis*. GC-rich motifs, commonly dominant in monocots, were the most frequent trinucleotide repeats in *Oryza* as the group GGC [11, 24, 28, 37] while the AAG group prevailed in *Arabidopsis* and GC-rich motifs were absent [24].

Overall, a major fraction of EST-SSRs were located in exons, an observation that seems consistent with EST-SSRs deriving from transcribed regions. Nevertheless, not every type of nucleotide repeat appeared in exons with equal probability. Di, tetra and pentanucleotide repeats were mostly concentrated in UTRs and, to a lesser extent, in other non-coding regions, whereas tri- and hexanucleotide repeats regularly occurred in exons. Since the frequency and distribution of the different SSR repeats and their motifs are function of the dynamics and history of genome evolution, the predominance of trinucleotide repeats in ESTs is attributed to selection against frameshift mutations caused by length variation in non-trinucleotide repeat motifs [12]. Large frequencies of dinucleotide repeats in UTRs and the prevalence of trinucleotide repeats in exons have been consistently reported in plant studies [28, 42]. Since EST sequences are derived from mRNA, the frequency of EST-SSRs located in non-coding regions might seem higher than expected. However, transcripts of unknown function with apparently little protein coding capacity are known to overlap with protein-coding regions and they are often distributed in intergenic regions [43].

Interestingly, trinucleotide repeats in *Oryza* were rich in GC motifs and more than 70 % of these GC-rich trinucleotides were related to exons. CCG repeats have been found to be involved in many gene functions as stress resistance, transcription regulation, or metabolic enzyme biosynthesis [28]. Trinucleotide repeats usually involve a moderate number of repeats because they do not perturb the reading frame but they may alter the stability of the quaternary structure of the resulting protein; this may result in low levels of polymorphism [44]. In contrast, dinucleotide repeats tend to display higher levels of variation as consequence of their association with UTRs and other non-coding regions [27, 45].

### EST-SSRs analysis from the IUCN genera

Overall, the frequencies of the various nucleotide repeats and motifs in IUCN genera were highly consistent with the results derived from the control genomes *Oryza* and *Arabidopsis*. Tri- and dinucleotide repeats accounted for more than 60 % of the total EST-SSRs, while tetra-, penta- and hexanucleotide repeats displayed lower frequencies. However, the abundance of the various types of repeat differed between groups. The latter, was expected because the SSRs distribution is a function of the dynamics and history of genome evolution [12]. Results from monocots and eudicots were highly consistent with the two control genomes and with previous findings in flowering plants where trinucleotide repeats were the most abundant motifs followed by dinucleotide repeats [24]. Similarly, the AG group was the commonest dinucleotide repeat, as it is typically the case in angiosperms [11, 12, 24, 37, 46]. The pattern seen in the trinucleotide motifs of IUCN genera agreed with what we found in *Oryza* and *Arabidopsis,* corroborating the high abundance of CG-rich motifs in monocots and the AAG group in dicots [11, 24, 28, 37]. In line with earlier studies, Acrogymnospermae revealed a higher proportion of hexanucleotide repeats, as well as dinucleotide repeats from the AT group when compared with monocots and eudicots [24, 46, 47]. Unfortunately, the four groups of non-vascular plants were represented by too few genera to allow generalizations.

Finally, in some genera (e.g. Taiwania and Urochloa) no Type I SSR was detected despite that the number of EST sequences in the data set seemed enough (2 624 and 2 207 respectively). The latter might be a consequence of the conservative criteria use in this study in an effort to increase the polymorphism of the detected ETS-SSRs [41]. To test the impact of the mining criteria a second SSR search, using more relaxed parameters, was carried out in the 64 genera with no Type I SSR and in ten randomly selected genera with Type I SSR. The SSR discovery was done using the default parameter suggested by QDD1, which is at least four repeats for each type of perfect SSRs. By doing so, four out of the 64 genera rendered SSRs, but only one SSR each one. Thus, it seems that in most of the cases the absence of output for the SSR search was largely caused by the filtering parameters instead of the searching criteria, indicating high rates of redundancy and/or short sequences in the input file. However, when the same test was performed in the ten randomly selected genera with Type I SSR the impact of relaxing the searching parameters was larger (see Additional file 3: Table S2). The number of EST-SSRs detected increased an average of 67.79 %, ranging from 34.78 % till 116 %. As expected, the higher impact was in di- and trinucleotide repeats. Therefore, in those cases when the number of EST sequences for the pet species is not very large, or the number of Type I SSR is too small, the parameters for SSR mining can be relaxed allowing the detection of a larger number of markers.

### Amplification and transferability of the EST-SSRs

Amplification success in this study was similar to values reported in some studies of EST-SSRs [48, 49] but lower than others [50, 51]. Unsuccessful primer amplification can be a consequence of non-transcribed introns located in the primer region [41]. Also, some of the EST-SSRs detected in our searches could actually belong to a different organism. As revealed by the analysis of *Arabidopsis* and *Oryza*, a portion of EST sequences did not find a match in their annotated genomes and might be a result of RNA contamination [17].

Given their association with conserved genome regions, EST-SSRs are often assumed to be less polymorphic than their genomic counterparts [9, 17, 52]. However, studies comparing both types of markers showed that this premise does not always hold true and similar levels of polymorphism have been found in anonymous SSRs *versus* EST-SSRs [18, 19]. Since only few individuals of each genus were selected to test the performance of our EST-SSRs, the levels of polymorphism detected in this study cannot be considered a general attribute of EST-SSRs. Saying so, our EST-SSRs showed acceptable levels of polymorphism within species, as well as divergence between species. The quality of the banding patterns was high, with clear peaks (except for the T1 pair), a flat baseline, and no null allele was detected. Cleaner profiles and lower frequencies of null alleles than those found with anonymous SSRs appear to be a general property of EST-SSRs [22, 51]. The lower levels of polymorphism usually attributed to EST-SSRs compared with anonymous SSRs may be compensated by their high rate of cross-species transferability [19, 29, 51], which has been reported not only among congenerics, but also across species of different genera [13]. Our results are highly congruent with the premise of high-transferability in EST-SSRs as all of the tested primers that successfully amplified in one species did the same in its counterpart and most of the loci displayed two or more alleles for the genera. Consequently, EST-SSRs arise as molecular markers with great potential for comparative studies among species.

### Use of EST-SSRs as molecular markers for studying threatened species

EST-SSRs can be used for essentially the same purposes as genomic SSRs but their link to translated regions offers a range of possibilities not usually available in anonymous SSRs. The function of EST-SSRs linked to coding regions can be identified by comparison with protein databases, with annotated genomes of closely related species or with model organisms such as *Arabidopsis* for eudicots and *Oryza* for monocots. By doing so, researchers interested in threatened species can go one step further in their studies and infer levels of functional genetic diversity [17, 25]. This topic has been largely disregarded due to the absence of well annotated genomes in non-model species like is the case in most threatened plant species. Therefore, the use of EST-SSRs for population studies will facilitate overcoming this issue. Furthermore, these markers can also be used in phylogenetic studies [26] and in comparative mapping studies thanks to their high cross-species transferability [13, 14].

It is often warned that population structure parameters estimated with EST-SSRs loci must be interpreted with caution as they may display a signature of selection [19]. However, this behavior is advantageous in studies targeting the so called “adaptive variation”, a topic of high relevance in conservation studies [1]. So far, studies trying to identify signatures of selection relied on the detection of outlier loci using putatively neutral markers. As mentioned before, EST-SSRs are associated with the transcribed region of the genome, thus they have a higher probability to be under selective pressures. Besides, for those markers showing a sign of selection a putative function can be deduced by comparing the sequence containing the SSR against publicly available databases as the non-redundant protein database from the NCBI. Interestingly, several studies reported that population structure measures derived from EST-SSRs were in agreement with those from anonymous SSRs, and only a small fraction of all genes might have experienced recent positive selection [22–24]. Therefore, the variation in the degree of neutrality of EST-SSRs allows to choose appropriate markers for several types of studies, from neutral and near-neutral loci for estimates of genetic drift or gene flow, to non-neutral ones for studying selection-related questions.

Our results derived from the control genomes suggest that conservation studies with an aim on functional variation and/or interested in detecting signatures of selection should focus on trinucleotide repeats because they are highly likely to be located within exons and are more abundant and more polymorphic than hexanucleotide repeats. Although dinucleotide repeats are mainly linked to non-coding regions and they are expected to behave as neutral markers they should not be rule out from conservation studies because they are known to be very polymorphic and our results show that they are mainly linked to UTRs, which are known to be involved in gene expression and other control functions [53]. Overall, we would recommend that, when using EST-SSRs, di- and trinucleotide repeats should be combined for a more comprehensive approach. This way trinucleotide repeats would cover the direct link with exons displaying a higher probability of being subjected to selection processes while dinucleotide repeats would offer larger levels of polymorphism and their probable neutral behavior will facilitate the inference of population structure measures non-biased by selection. Moreover, since all EST-SSRs are associated with the transcribed region of the genome they can be used to target functional genetic diversity in threatened species. Besides, EST sequences containing SSRs can be cross-referenced with annotated genomes for sequence similarity and gene discovery.

## Conclusions

In summary, this study represents the first large-scale attempt to assess the potential of publicly accessible EST databases as a source for SSRs discovery in threatened plants. Our results highly support the use of existing EST databases for SSRs discovery in non-model plants as a bench tool for evolutionary and/or conservation studies of geneticists and molecular ecologists. With this approach, we identified a very large number of ready-to-test EST-SSRs in most of the IUCN plant genera used in this study. Our tests indicate that these SSRs can show high transferability rate among species. Therefore, the set of loci presented here possibly has a very large number of potential target species. Moreover, a portion of our loci might be functional markers providing relevant information about “adaptive variation”, which is a subject of high interest in conservation studies. In fact, the variation in the degree of neutrality of EST-SSRs allows to select markers that may be appropriate for various research topics. Developing molecular markers for the species of interest is one of the most frequent rate-limiting steps in population genetic studies. In this regard, our results show that EST databases are a valuable and suitable source for SSRs discovery. Unlike the demanding classical procedure for genomic SSR development, a set of EST-SSRs with primers can be produced in a couple of days at no additional cost once the EST database has been accessed.

### Availability of supporting data

The data sets supporting the results of this article (i.e. all the EST-SSRs, with their respective primers, developed in the present study for 193 plant genera from the IUCN Red List and for *Arabidopsis*) are publicly available in the Dryad database with doi:10.5061/dryad.63h33 (instructions for the database in Additional file 4).

## Additional files

Additional file 1:
**Programming scripts.** (DOCX 20 kb) Additional file 2: Table S1. List of the IUCN plant genera mined for EST-SSRs with raw results. Number of species for each genera within each IUCN category (EX = extinct, EW = extinct in the wild, CR = critically endangered, EN = endangered, VU = vulnerable, NT = near threatened, LC = least concern, DD =data deficient). N EST, number of EST sequences downloaded for each genera; di-, tri-, tetra-, penta- and hexa- denotes type of SSRs and total indicates the total number of SSRs with primer information found for each genera. (DOCX 52 kb) Additional file 3: Table S2. Impact of the mining criteria (Type I or relaxed parameters) in the total number of EST-SSRs detected in ten randomly selected genera. (DOCX 13 kb) Additional file 4:
**Instructions for the database use.** (DOCX 147 kb)

## Abbreviations

EST: Expressed Sequence Tags
SSR: Simple Sequence Repeats
EST-SSR: SSR mined form EST sequences
NGS: Next Generation Sequencing
IUCN: International Union for the Conservation of the Nature
NCBI: National Center for Biotechnology Information
BLAST: Basic Local Alignment Search Tool
UTR: untranslated region
