# Supplementary material for: Mining microsatellite markers from public expressed sequence tags databases for the study of threatened plants
Source: BMC Genomics. 2015 Oct 13;16:781. doi: 10.1186/s12864-015-2031-1 (PMC4603344; doi:10.1186/s12864-015-2031-1)
Supplement: Additional file 1: — Programming scripts. (DOCX 20 kb) [file 12864_2015_2031_MOESM1_ESM.docx]

**Programming scripts.**

The following scripts were used for inferring the occurrence and frequency of the SSR motifs in the analyzed plant genera using MATLAB and Statistics Toolbox 2013a (MathWorks Inc., MA, US). A, b, c and d belong to four different files. File a is the main script while b, c and d are functions called by that main script. File a (process folder) searches in the specified path all the .tbl files (i.e. output files from QDD1) and defines structures to store the relevant data contained in these files. Then, it calls function b (treat_tbl) for each of the found files. Treat_tbl parses the different .tbl files looking for the markers labelled as “best” by QDD1 and extract the information regarding the number of each type of repeat and the different motifs. Functions c (num2bases) and d (bases2num) support the execution of function b. Finally, all the information obtained from each .tbl file is saved at the species level, at the group level (e.g. taxonomic group Florideophyceae), as well as for the total data in the specified path. Please, note that no scripts are detailed for the SSR search because for this step was used the friendly version of QDD1.

1. Process folder

%% scan folders, process files

clear

% [status,list]=system('find . -name "*.tbl"');

[status,list]=system('dir *.tbl /b/s');

%separators = find(strcmp('/n',list));

list_cell = {};

counter =1;

i = 1;

start = [];

while i <=length(list)

if list(i) == 'C' && list(i+1) == ':'

start (counter)= i;

counter = counter +1;

end

i=i+1;

end

start(counter) = length(list);

for i=1:counter -1

if list(start(i+1)-1) =='l'

list_cell{i,1} = list(start(i):start(i+1)-1);

else

list_cell{i,1} = list(start(i):start(i+1)-2);

end

end

current_path = pwd;

mat_reps = [];

cell_species = {};

cell_group = {};

oldPathName = 'old';

for i=1:length(list_cell)

break_point = find(list_cell{i} =='\',1,'last')+1;

FileName = list_cell{i}(break_point:end);

PathName = list_cell{i}(1:break_point-1);

nName = PathName(find(PathName=='_',1,'last')+1:end-1);

cd(PathName);

if ~strcmp(PathName,oldPathName)

if exist('total_nucleotides','var')

cd(current_path)

cd(oldPathName)

group_name = nName;

bases = [];

[ii,jj,ss] = find(total_nucleotides);

ii = num2bases(ii);

cumulated_SSRs = mat2dataset(ss,'ObsNames',ii);

export(cumulated_SSRs,'file',[group_name,'_SSRs.csv'],'Delimiter','comma');

cd(current_path)

cd(PathName)

else

total_nucleotides = [];

end

end

output_File = [FileName(1:find(FileName=='.',1,'last')-1),'.fas'];

if ~exist(output_File)

fprintf('Processing file %s.\n.',list_cell{i});

[species, reps, nucleotides] = treat_tbls(FileName);

else

fprintf('File %s already processed.\n',list_cell{i});

end

mat_reps = [mat_reps;reps];

cell_species = [cell_species;[nName,'_',species]];

cell_group = [cell_group;nName];

if isempty(nucleotides)

elseif isempty(total_nucleotides)

total_nucleotides = nucleotides;

else

total_nucleotides = total_nucleotides + nucleotides;

end

oldPathName = PathName;

cd(current_path)

end

%%

cd(current_path)

cd(oldPathName)

if exist('total_nucleotides','var')

bases = [];

group_name = nName;

[ii,jj,ss] = find(total_nucleotides);

ii = num2bases(ii);

cumulated_SSRs = mat2dataset(ss,'ObsNames',ii);

export(cumulated_SSRs,'file',[group_name,'_SSRs.csv'],'Delimiter','comma');

end

cd(current_path)

summarized = mat2dataset(mat_reps);

summarized.Properties.ObsNames = cell_species;

summarized.Properties.VarNames = {'a_','Di','Tri','Tetra','Penta','Hexa'};

summarized.a_ = [];

summarized.Group = cell_group;

summarized.species = cell_species;

sortrows(summarized,{'Group' 'species'});

summarized.species = [];

export(summarized,'file','summarized.csv','Delimiter','comma');

1. Treat_tbls

function [species, reps, nucleotides] = treat_tbls(path_input)

output_file = path_input(1:find(path_input=='_',1,'first')-1);

fileID = fopen(path_input);

headers = textscan(fileID,'%s',29,'delimiter',';','EmptyValue',0);

C= textscan(fileID,'%s %d %d %f %f %f %s %s %d %d %d %d %f %f %f %f %d %d %d %d %d %d %d %d %d %d %d %s %s %s','delimiter',';');

fclose(fileID);

cmotif = C{28};

clength_bp = num2cell(C{26});

cprod1 = num2cell(C{2});

cprod2 = num2cell(C{3});

cprimerl = C{7};

cprimerl2 = num2cell(C{9});

cprimerr = C{8};

cprimerr2= num2cell(C{11});

cID = C{1};

clabel = C{30};

clength_rep = num2cell(C{27});

lengthmotif = cellfun('length',cmotif);

C_total = [cmotif';clength_bp';cprod1';cprod2';cprimerl';cprimerl2';cprimerr';cprimerr2';cID';num2cell(lengthmotif)';clength_rep']';

best_rows = strcmp('best',clabel) & cell2mat(clength_bp)>=20;

if ~any(best_rows)

fprintf('No entry in %s matched the requirements.\n',path_input)

fid=fopen([output_file,'_failed'],'w');

fclose(fid);

species = [output_file,'_failed'];

reps = zeros(1,6);

nucleotides = [];

return

end

C = C_total(best_rows,:);

C = sortrows(C,[10,1,2]);

output_cell = cell(sum(best_rows)+1,7);

output_cell(1,:) = {'Motif' 'nreps' 'PCR_prod' 'Primer_L_pos' 'Primer_R_pos' 'ID' 'length_bp'};

for i =1:sum(best_rows)

output_cell(i+1,:) = {C{i,1} C{i,11} [num2str(C{i,3}),'-',num2str(C{i,4})]...

[C{i,5},' (',num2str(C{i,6}),')'] [C{i,7},' (',num2str(C{i,8}),')'] C{i,9} C{i,2}};

combined (i)= {[C{i,1},'_',num2str(C{i,11})]};

end

detailed_table = cell2dataset(output_cell);

export(detailed_table,'file',[output_file,'_table_detailed.csv'],'Delimiter','comma');

mot_nominal = nominal(C(:,1));

[table,~,~,labels] = crosstab(mot_nominal,cell2mat(C(:,11)));

[rows,cols] = size(labels);

for i=1:rows

labels(i,2) = {['rep_',labels{i,2}]};

end

table2 = mat2dataset (table);

[m n]=size(table);

table2.Properties.ObsNames = labels(1:m,1);

for i =1:length(table2(:,1))

nelems(i) = length(table2.Properties.ObsNames{i});

end

table2.Properties.VarNames = labels(1:n,2);

table2.lengthmotif = nelems';

table2.rowsum = sum(table,2);

nreps = tabulate(nelems');

for i =1:length(table2(:,1))

nclase(i) = nreps(find(nelems(i) == nreps(:,1),1,'first'),1);

end

table2.nclass = nclase';

table2.nclass2 = zeros(size(table2.nclass));

ngroups = [];

for i =1:max(nreps(:,1))

temp = sum(table2.rowsum(table2.nclass == nreps(i,1)));

table2.nclass2(table2.nclass == nreps(i,1)) = temp;

ngroups(i) = temp;

end

table2.motif = table2.Properties.ObsNames;

table2 = sortrows(table2,{'lengthmotif','motif'});

table3 = table2;

table2.motif = [];

table2.nclass = [];

table2.rowsum = [];

table2.lengthmotif = [];

export(table2,'file',[output_file,'_table_reps.csv'],'Delimiter','comma');

species = output_file;

reps = zeros(1,6);

[m n]=size(ngroups);

reps(1:n) = ngroups;

%% table only with observations >=2 and only total n reps

table3 = mat2dataset([double(table3.rowsum)';double(table3.lengthmotif)']',...

'ObsNames',table2.Properties.ObsNames,'VarNames',{'nreps' 'lengthmotif' });

table3(table3.nreps<2,:) = [];

table3 = sortrows(table3,{'lengthmotif','nreps'},{'ascend','descend'});

cols = {};

for ii = 1 : length(table3.lengthmotif);

switch table3.lengthmotif(ii)

case 2

cols{ii} = 'b';

case 3

cols{ii} = 'r';

case 4

cols{ii} = 'k';

case 5

cols{ii} = 'g';

case 6

cols{ii} = 'm';

end

end

table3.lengthmotif = [];

export(table3,'file',[output_file,'_table_totalreps.csv'],'Delimiter','comma');

%% codify motif

codes = [];

values = [];

for i=1:length(table3.nreps);

codes(i) = bases2num(table3.Properties.ObsNames{i});

values(i) = table3.nreps(i);

end

nucleotides = sparse(codes,ones(size(codes)),values,444444,4^4);

%% table only with number of di, tri, tetra-... nucleotides

names_string = {'DNRs' 'TRNs' 'TTNs' 'PNRs' 'HNRs'};

table4 = mat2dataset(reps(2:end)','ObsNames', names_string,'VarNames','nreps');

export(table4,'file',[output_file,'_table_classification.csv'],'Delimiter','comma');

end

1. num2bases

function base = num2bases(code_in)

len = length(code_in);

base = cell(size(code_in));

for i =1:len

code = code_in(i);

code = num2str(code);

code(code == '1') = 'a';

code(code == '2') = 'c';

code(code == '3') = 'g';

code(code == '4') = 't';

base{i} = code;

end

end

1. bases2num

function code = bases2num(base)

base(base == 'a') = '1';

base(base == 'c') = '2';

base(base == 'g') = '3';

base(base == 't') = '4';

code = str2num(base);

end
