# Supplementary material for: Mining microsatellite markers from public expressed sequence tags databases for the study of threatened plants
Source: BMC Genomics. 2015 Oct 13;16:781. doi: 10.1186/s12864-015-2031-1 (PMC4603344; doi:10.1186/s12864-015-2031-1)
Supplement: Additional file 2: Table S1. — List of the IUCN plant genera mined for EST-SSRs with raw results. Number of species for each genera within each IUCN category (EX = extinct, EW = extinct in the wild, CR = critically endangered, EN = endangered, VU = vulnerable, NT = near threatened, LC = least concern, DD =data deficient). N EST, number of EST sequences downloaded for each genera; di-, tri-, tetra-, penta- and hexa- denotes type of SSRs and total indicates the total number of SSRs with primer information found for each genera. (DOCX 52 kb) [file 12864_2015_2031_MOESM2_ESM.docx]

| **Taxonomy** | | | | **IUCN records** | | | | | | | | |  | **EST-SSRs** | | | | | |
| --- | --- | --- | --- | --- | --- | --- | --- | --- | --- | --- | --- | --- | --- | --- | --- | --- | --- | --- | --- |
| **Group** | **Class** | **Family** | **Genus** | **EX** | **EW** | **CR** | **EN** | **VU** | **NT** | **LC** | **DD** | **tot** | **n EST** | **di** | **tri** | **tetra** | **penta** | **hexa** | **Tot** |
| **Rhodophyta** | **Florideophyceae** | **Gigartinaceae** | ***Chondrus*** |  |  |  |  |  |  |  | 2 | 2 | 4114 |  |  |  |  | 3 | 3 |
|  |  | **Gracilariaceae** | ***Gracilaria*** |  |  | 1 |  |  |  |  | 1 | 2 | 12531 | 2 | 10 | 2 | 1 | 7 | 22 |
| **Charophyta** | **Charophyceae** | **Characeae** | ***Nitella*** |  |  |  |  |  |  | 7 |  | 7 | 88280 | 16 | 77 | 39 | 38 | 30 | 200 |
| **Monilophyta** | **Osmundiopsida** | **Osmundaceae** | ***Osmunda*** |  |  |  |  |  |  | 1 |  | 1 | 2684 | 1 | 3 | 1 |  |  | 5 |
|  | **Polypodiopsida** | **Adiantaceae** | ***Adiantum*** | 1 |  |  | 2 |  |  | 1 |  | 4 | 30540 | 122 | 16 | 3 | 1 | 4 | 146 |
|  |  | **Azollaceae** | ***Azolla*** |  |  |  |  |  |  | 2 |  | 2 | 6 |  |  |  |  |  | 0 |
|  |  | **Marsileaceae** | ***Marsilea*** |  |  |  | 1 | 2 |  | 13 |  | 16 | 61 |  |  |  |  |  | 0 |
|  |  | **Pteridaceae** | ***Ceratopteris*** |  |  |  |  |  |  | 2 |  | 2 | 5125 | 7 | 2 |  | 1 | 2 | 12 |
| **Lycopodiophyta** | **Isoetopsida** | **Isotaceae** | ***Isoetes*** |  |  | 8 | 4 | 2 | 3 | 5 | 6 | 28 | 338 |  |  |  |  | 1 | 1 |
|  | **Lycopodiopsida** | **Lycopodiaceae** | ***Huperzia*** |  |  |  | 2 | 8 | 1 | 1 |  | 12 | 3451 | 1 | 9 | 2 | 1 | 10 | 23 |
|  | **Selaginellopsida** | **Selaginellaceae** | ***Selaginella*** |  |  |  | 1 |  | 1 | 2 |  | 4 | 97503 | 19 | 113 | 13 | 6 | 15 | 166 |
| **Acrogymnospermae** | **Pinopsida** | **Araucariacea** | ***Araucaria*** |  |  | 2 | 5 | 6 |  |  |  | 13 | 10 |  |  |  |  |  | 0 |
|  |  | **Cupressaceae** | ***Chamaecyparis*** |  |  |  | 1 | 1 |  |  |  | 2 | 6702 | 0 | 2 | 1 | 1 | 2 | 6 |
|  |  |  | ***Taiwania*** |  |  |  |  | 1 |  |  |  | 1 | 2624 |  |  |  |  |  | 0 |
|  |  |  | ***Cunninghamia*** |  |  |  |  | 1 |  | 1 |  | 2 | 444 |  | 1 |  |  |  | 1 |
|  |  |  | ***Cryptomeria*** |  |  |  |  |  | 1 |  |  | 1 | 57653 | 1 | 4 | 1 | 2 | 4 | 12 |
|  |  | **Pinaceae** | ***Picea*** |  |  | 2 | 5 | 5 | 2 | 22 |  | 36 | 546864 | 55 | 44 | 12 | 23 | 51 | 185 |
|  |  |  | ***Pinus*** |  |  | 1 | 2 | 7 | 15 | 85 |  | 110 | 476951 | 38 | 29 | 6 | 15 | 64 | 152 |
|  |  |  | ***Pseudotsuga*** |  |  |  |  | 2 | 1 | 1 |  | 4 | 18142 | 1 | 1 |  | 4 | 8 | 14 |
|  |  |  | ***Abies*** |  |  | 4 | 4 | 4 | 3 | 30 | 1 | 46 | 2806 |  |  |  |  | 2 | 2 |
|  |  |  | ***Larix*** |  |  |  |  | 1 |  | 9 |  | 10 | 965 |  | 2 |  |  | 2 | 4 |
|  |  | **Podocarpaceae** | ***Podocarpus*** |  |  | 3 | 14 | 8 | 11 | 51 | 11 | 98 | 677 | 3 |  |  | 3 | 1 | 7 |
|  |  | **Sciadopitytaceae** | ***Sciadopitys*** |  |  |  |  | 1 |  |  |  | 1 | 11 | 1 | 2 |  |  |  | 3 |
|  |  | **Taxaceae** | ***Taxus*** |  |  | 1 | 2 | 1 | 2 | 4 |  | 10 | 492 |  |  |  |  |  | 0 |
|  | **Cycadiopsida** | **Cycadaceae** | ***Cycas*** |  |  | 11 | 16 | 30 | 24 | 15 | 2 | 98 | 21997 | 10 | 1 | 2 | 2 | 1 | 16 |
|  |  | **Zamiaceae** | ***Zamia*** |  |  | 15 | 13 | 11 | 20 | 1 |  | 60 | 20677 | 13 | 22 | 1 | 3 | 7 | 46 |
|  | **Gnetopsida** | **Ephedraceae** | ***Ephedra*** |  |  |  |  |  | 1 | 51 | 4 | 56 | 4981 |  | 4 | 1 | 1 | 13 | 19 |
|  |  | **Gnetaceae** | ***Gnetum*** |  |  |  | 1 | 3 | 6 | 25 | 6 | 41 | 10724 | 6 | 26 | 5 | 3 | 32 | 72 |
|  | **Ginkgopsida** | **Ginkgoaceae** | ***Ginkgo*** |  |  |  | 1 |  |  |  |  | 1 | 21590 | 16 | 7 | 1 | 1 | 6 | 31 |
| **Magnoliidae** | **Magnoliidae** | **Magnoliaceae** | ***Lirodendron*** |  |  |  |  |  | 1 |  |  | 1 | 24663 | 84 | 32 | 4 | 1 | 8 | 129 |
|  |  | **Lauraceae** | ***Persea*** |  |  |  | 1 | 11 | 3 | 1 |  | 16 | 16558 | 56 | 14 | 2 | 6 | 8 | 86 |
|  |  | **Aristolochiaceae** | ***Aristolochia*** |  |  | 2 | 3 | 6 |  |  |  | 11 | 16451 | 27 | 32 | 2 | 2 | 4 | 67 |
|  |  |  | ***Saruma*** |  |  |  | 1 |  |  |  |  | 1 | 10274 | 26 | 11 | 2 | 0 | 10 | 49 |
|  |  |  | ***Asarum*** |  |  |  |  | 2 |  |  |  | 2 | 623 |  |  | 1 |  |  | 1 |
| **Monocotyledoneae** | **Liliopsida** | **Acoraceae** | ***Acorus*** |  |  |  |  |  |  | 2 |  | 2 | 9733 | 26 | 19 | 3 | 1 | 3 | 52 |
|  |  | **Alliaceae** | ***Allium*** |  |  | 1 | 1 | 4 | 2 | 11 | 37 | 56 | 41796 | 17 | 16 | 2 | 3 | 13 | 51 |
|  |  | **Amaryllidaceae** | ***Narcissus*** |  |  |  | 5 |  |  | 6 | 1 | 12 | 1 |  |  |  |  |  | 0 |
|  |  | **Araceae** | ***Zantedeschia*** |  |  |  |  |  |  | 2 |  | 2 | 4283 | 9 | 2 |  |  | 1 | 12 |
|  |  |  | ***Scindapsus*** |  |  |  |  |  |  | 1 |  | 1 | 18 |  |  |  |  |  | 0 |
|  |  |  | ***Colocasia*** |  |  |  |  |  |  | 2 |  | 2 | 9 |  |  |  |  |  | 0 |
|  |  |  | ***Landoltia*** |  |  |  |  |  |  | 1 |  | 1 | 154 |  |  |  |  |  | 0 |
|  |  |  | ***Wolffia*** |  |  |  |  |  |  | 3 |  | 3 | 1827 |  | 1 |  |  |  | 1 |
|  |  |  | ***Lemna*** |  |  |  | 1 |  |  | 6 |  | 7 | 16 |  |  |  |  |  | 0 |
|  |  | **Asparagaceae** | ***Asparagus*** |  |  |  | 3 | 2 |  | 2 | 3 | 10 | 8519 | 16 | 20 | 1 | 2 | 4 | 43 |
|  |  | **Agavaceae** | ***Agave*** |  |  |  |  |  |  | 1 |  | 1 | 4 |  |  |  |  |  | 0 |
|  |  | **Bromeliaceae** | ***Guzmania*** |  |  | 4 | 10 | 19 | 5 | 6 | 1 | 45 | 1 |  |  |  |  |  | 0 |
|  |  | **Costaceae** | ***Costus*** |  |  |  |  | 2 |  | 1 |  | 3 | 1221 | 1 |  |  |  | 1 | 2 |
|  |  | **Dioscoreaceae** | ***Dioscorea*** |  |  | 1 | 2 | 2 | 1 | 8 | 2 | 16 | 44169 | 14 | 40 | 6 | 6 | 16 | 82 |
|  |  | **Poaceae** | ***Oryza*** |  |  |  | 2 |  |  | 2 |  | 4 | 1342281 | 101 | 315 | 43 | 60 | 62 | 521 |
|  |  |  | ***Panicum*** |  |  | 2 | 1 |  |  | 6 |  | 9 | 720785 | 63 | 205 | 39 | 56 | 96 | 459 |
|  |  |  | ***Saccharum*** |  |  |  |  |  |  | 3 |  | 3 | 284272 | 64 | 178 | 33 | 50 | 92 | 417 |
|  |  |  | ***Sorghum*** |  |  |  |  |  |  | 3 |  | 3 | 232681 | 59 | 174 | 45 | 57 | 61 | 396 |
|  |  |  | ***Festuca*** |  |  |  | 1 | 4 | 2 | 5 | 1 | 13 | 139903 | 24 | 74 | 27 | 16 | 24 | 165 |
|  |  |  | ***Avena*** |  |  |  |  |  |  | 1 | 1 | 2 | 79586 | 26 | 81 | 28 | 17 | 29 | 181 |
|  |  |  | ***Setaria*** |  |  |  |  |  |  | 2 |  | 2 | 66027 | 7 | 17 | 4 | 11 | 3 | 42 |
|  |  |  | ***Elymus*** |  |  |  |  |  |  | 1 |  | 1 | 27891 | 13 | 26 | 4 | 8 | 10 | 61 |
|  |  |  | ***Cenchrus*** |  |  | 1 |  |  |  |  |  | 1 | 21708 | 7 | 16 | 5 | 3 | 3 | 34 |
|  |  |  | ***Agrostis*** |  |  |  | 1 |  | 2 | 4 | 1 | 8 | 21655 | 4 | 5 | 2 | 2 | 3 | 16 |
|  |  |  | ***Eragrostis*** |  |  |  |  |  |  | 11 |  | 11 | 15898 | 6 | 5 | 7 | 2 | 0 | 20 |
|  |  |  | ***Puccinellia*** |  |  | 1 |  | 1 |  |  |  | 2 | 15037 | 6 | 18 | 3 | 5 | 2 | 34 |
|  |  |  | ***Urochloa*** |  |  |  |  |  |  | 1 |  | 1 | 2207 |  |  |  |  |  | 0 |
|  |  |  | ***Eleusine*** |  |  |  |  |  |  | 1 |  | 1 | 1934 | 1 | 3 |  |  | 1 | 5 |
|  |  |  | ***Bambusa*** |  |  |  |  |  |  | 1 |  | 1 | 514 | 4 |  |  |  |  | 4 |
|  |  |  | ***Brachiaria*** |  |  |  |  |  |  | 6 |  | 6 | 41 | 1 |  |  |  |  | 1 |
|  |  |  | ***Vetiveria*** |  |  |  |  |  |  | 1 |  | 1 | 32 | 1 |  |  |  |  | 1 |
|  |  |  | ***Poa*** |  |  |  | 1 |  |  | 8 | 1 | 10 | 30 |  |  |  |  |  | 0 |
|  |  |  | ***Agropyron*** |  |  |  | 2 |  | 1 |  |  | 3 | 16 |  |  |  |  |  | 0 |
|  |  |  | ***Paspalum*** |  |  |  | 2 | 1 | 6 |  |  | 9 | 7 |  |  |  |  |  | 0 |
|  |  |  | ***Digitaria*** |  |  |  |  |  |  | 4 | 3 | 7 | 1 |  |  |  |  |  | 0 |
|  |  |  | ***Phalaris*** |  |  |  |  | 1 |  | 1 |  | 2 | 1 |  |  |  |  |  | 0 |
|  |  | **Hydrocharitaceae** | ***Hydrilla*** |  |  |  |  |  |  | 1 |  | 1 | 70 |  |  |  |  |  | 0 |
|  |  | **Iridaceae** | ***Crocus*** |  |  |  |  | 2 | 1 |  |  | 3 | 6767 | 3 | 5 | 3 | 1 | 3 | 15 |
|  |  |  | ***Iris*** |  |  | 1 |  |  |  |  | 2 | 3 | 6745 | 31 | 41 | 6 | 2 | 8 | 88 |
|  |  | **Liliaceae** | ***Lilium*** |  |  |  |  | 1 |  | 1 | 1 | 3 | 3906 |  |  |  |  | 2 | 2 |
|  |  |  | ***Fritillaria*** |  |  |  | 4 | 2 |  |  | 3 | 9 | 2158 | 2 | 2 |  |  | 2 | 6 |
|  |  |  | ***Tulipa*** |  |  |  | 4 | 2 |  |  | 3 | 9 | 76 |  |  |  |  |  | 0 |
|  |  | **Orchidaceae** | ***Dendrobium*** |  |  | 3 | 8 | 1 |  | 7 | 3 | 22 | 16183 | 31 | 18 | 3 | 2 | 9 | 63 |
|  |  |  | ***Phalaenopsis*** |  |  | 2 | 1 | 1 |  |  |  | 4 | 8066 | 3 | 5 | 1 |  | 2 | 11 |
|  |  |  | ***Vanda*** |  |  |  | 2 |  |  | 1 |  | 3 | 2271 | 5 | 5 | 1 |  | 1 | 12 |
|  |  |  | ***Oncidium*** |  |  |  |  |  |  | 1 |  | 1 | 3138 | 6 | 5 |  | 1 | 2 | 14 |
|  |  |  | ***Cymbidium*** |  |  |  | 2 |  |  |  | 1 | 3 | 78 |  |  |  |  |  | 0 |
|  |  |  | ***Calanthe*** |  |  |  | 1 | 3 |  |  | 1 | 5 | 66 |  |  |  |  |  | 0 |
|  |  |  | ***Vanilla*** |  |  |  | 1 |  |  | 1 |  | 2 | 31 |  |  |  |  |  | 0 |
|  |  |  | ***Anoectochilus*** |  |  |  | 1 | 1 |  |  | 1 | 3 | 7 |  |  |  |  |  | 0 |
|  |  | **Arecaceae** | ***Calamus*** |  |  | 4 |  | 1 | 1 | 10 | 2 | 18 | 2528 | 4 | 6 |  | 2 | 3 | 15 |
|  |  |  | ***Metroxylon*** |  |  |  |  | 1 |  | 1 |  | 2 | 412 |  |  |  |  |  | 0 |
|  |  |  | ***Phoenix*** |  |  |  |  |  | 3 |  |  | 3 | 411 | 4 | 5 | 2 | 1 | 1 | 13 |
|  |  | **Pandaceae** | ***Pandanus*** |  |  | 5 | 1 | 12 | 5 | 3 | 2 | 28 | 977 | 4 | 1 |  |  | 1 | 6 |
|  |  | **Posidoniaceae** | ***Posidonia*** |  |  |  |  | 1 | 1 | 6 |  | 8 | 5668 | 8 | 16 | 1 |  | 2 | 27 |
|  |  | **Typhaceae** | ***Typha*** |  |  |  |  |  |  | 5 |  | 5 | 133 |  |  |  |  |  | 0 |
|  |  | **Zingiberaceae** | ***Zingiber*** |  |  |  | 1 | 1 |  | 5 | 1 | 8 | 38186 | 21 | 48 | 13 | 7 | 22 | 111 |
|  |  | **Zosteraceae** | ***Zostera*** |  |  |  | 2 | 2 | 2 | 8 |  | 14 | 18345 | 6 | 23 | 14 | 8 | 14 | 65 |
| **Eudicotyledoneae** | **Magnoliopsida** | **Acanthaceae** | ***Acanthus*** |  |  |  |  | 1 |  | 4 |  | 5 | 628 |  | 1 |  |  |  | 1 |
|  |  | **Actinidiaceae** | ***Actinidia*** |  |  |  | 1 | 7 |  |  |  | 8 | 132593 | 278 | 54 | 10 | 13 | 55 | 410 |
|  |  | **Aizoaceae** | ***Sesuvium*** |  |  |  |  |  |  | 1 |  | 1 | 38 |  |  |  |  |  | 0 |
|  |  | **Anacardiaceae** | ***Mangifera*** |  | 2 | 1 | 7 | 15 |  | 6 | 8 | 39 | 1665 | 0 | 1 | 0 | 0 | 0 | 1 |
|  |  |  | ***Pistacia*** |  |  |  |  | 2 | 2 | 1 |  | 5 | 1299 | 0 | 1 | 0 | 0 | 3 | 4 |
|  |  | **Apocynaceae** | ***Wrightia*** |  |  |  |  | 3 |  | 1 |  | 4 | 16431 | 25 | 26 | 3 | 2 | 7 | 63 |
|  |  | **Araliaceae** | ***Panax*** |  |  |  | 1 |  |  |  |  | 1 | 22225 | 31 | 32 | 11 | 14 | 28 | 116 |
|  |  |  | ***Eleutherococcus*** |  |  |  | 5 |  |  |  |  | 5 | 464 |  |  |  |  |  | 0 |
|  |  | **Avicenniaceae** | ***Avicennia*** |  |  |  |  | 4 |  | 5 |  | 9 | 1893 | 1 | 2 | 0 | 0 | 0 | 3 |
|  |  | **Berberidaceae** | ***Berberis*** |  |  | 2 | 2 | 9 | 2 | 2 | 14 | 31 | 10120 | 9 | 27 | 5 | 12 | 18 | 71 |
|  |  |  | ***Epimedium*** |  |  |  |  |  |  | 6 |  | 6 | 4983 | 6 | 16 | 7 | 2 | 9 | 40 |
|  |  | **Betulaceae** | ***Alnus*** |  |  |  |  |  | 1 | 2 |  | 3 | 32544 | 63 | 33 | 0 | 2 | 16 | 114 |
|  |  |  | ***Betula*** |  | 1 | 3 | 2 | 2 |  | 3 |  | 11 | 5910 | 9 | 4 | 0 | 2 | 0 | 15 |
|  |  |  | ***Corylus*** |  |  | 1 |  |  |  | 1 |  | 2 | 3 |  |  |  |  |  | 0 |
|  |  | **Boraginaceae** | ***Onosma*** |  |  | 1 |  |  |  |  | 1 | 2 | 78 |  |  |  |  |  | 0 |
|  |  | **Burseraceae** | ***Commiphora*** |  |  |  |  | 3 | 16 |  | 1 | 20 | 81 | 1 | 0 | 0 | 0 | 0 | 1 |
|  |  | **Cactaceae** | ***Opuntia*** |  |  |  |  |  |  | 8 |  | 8 | 451 | 1 | 1 | 0 | 0 | 1 | 3 |
|  |  | **Cleomaceae** | ***Cleome*** |  |  |  |  |  |  | 1 |  | 1 | 4677 | 20 | 15 | 0 | 1 | 6 | 42 |
|  |  | **Caricaceae** | ***Carica*** |  |  |  | 2 | 2 | 2 |  |  | 6 | 77393 | 57 | 36 | 1 | 4 | 15 | 113 |
|  |  | **Caryocaraceae** | ***Caryocar*** |  |  |  | 2 | 1 |  |  |  | 3 | 958 | 0 | 1 | 0 | 0 | 0 | 1 |
|  |  | **Caryophyllaceae** | ***Silene*** |  |  | 2 | 6 | 2 | 3 | 1 |  | 14 | 3849 | 4 | 10 | 3 | 1 | 5 | 23 |
|  |  |  | ***Dianthus*** |  |  | 1 | 1 | 1 | 1 | 1 | 3 | 8 | 23276 | 4 | 86 | 8 | 13 | 27 | 138 |
|  |  |  | ***Gypsophila*** |  |  |  | 1 |  |  |  |  | 1 | 148 | 0 | 0 | 0 | 0 | 1 | 1 |
|  |  | **Celastraceae** | ***Euonymus*** |  |  | 1 | 4 | 4 | 2 | 5 |  | 16 | 51380 | 17 | 15 | 3 | 1 | 13 | 49 |
|  |  |  | ***Catha*** |  |  |  |  |  |  | 1 |  | 1 | 445 | 23 | 7 | 2 | 0 | 3 | 35 |
|  |  | **Amaranthaceae** | ***Beta*** |  |  | 1 |  | 1 |  |  | 1 | 3 | 29830 | 6 | 24 | 1 | 1 | 33 | 65 |
|  |  |  | ***Suaeda*** |  |  |  |  |  |  | 1 |  | 1 | 1703 | 0 | 4 | 1 | 0 | 3 | 8 |
|  |  |  | ***Salicornia*** |  |  |  | 1 |  |  |  |  | 1 | 1432 | 0 | 1 | 0 | 0 | 1 | 2 |
|  |  |  | ***Atriplex*** |  |  | 1 |  |  |  |  |  | 1 | 519 | 0 | 1 | 1 | 0 | 1 | 3 |
|  |  | **Cistaceae** | ***Cistus*** |  |  |  | 1 |  |  |  | 1 | 2 | 2048 | 0 | 1 | 1 | 0 | 0 | 2 |
|  |  | **Combretaceae** | ***Terminalia*** |  | 1 |  | 6 | 12 | 1 |  | 1 | 21 | 9 |  |  |  |  |  | 0 |
|  |  | **Asteraceae** | ***Lactuca*** |  |  |  | 1 | 2 | 2 | 3 | 3 | 11 | 226709 | 84 | 213 | 23 | 32 | 138 | 490 |
|  |  |  | ***Artemisia*** |  |  | 1 | 1 | 1 | 1 | 2 | 1 | 7 | 95480 | 12 | 40 | 6 | 9 | 28 | 95 |
|  |  |  | ***Centaurea*** | 1 |  | 3 | 4 | 9 | 3 |  | 3 | 23 | 85293 | 24 | 146 | 33 | 26 | 77 | 306 |
|  |  |  | ***Carthamus*** |  |  |  |  |  |  | 1 |  | 1 | 41584 | 30 | 96 | 21 | 9 | 29 | 185 |
|  |  |  | ***Barnadesia*** |  |  |  | 1 |  |  | 1 |  | 2 | 28483 | 38 | 34 | 2 | 2 | 14 | 90 |
|  |  |  | ***Senecio*** |  | 1 | 2 | 1 | 1 |  | 4 | 2 | 11 | 10219 | 2 | 9 | 3 | 2 | 13 | 29 |
|  |  |  | ***Stevia*** |  |  |  | 1 | 2 |  |  | 2 | 5 | 5548 | 0 | 6 | 0 | 0 | 6 | 12 |
|  |  |  | ***Vernonia*** | 1 |  |  | 1 | 3 |  |  | 1 | 6 | 1325 | 2 | 1 | 1 | 0 | 2 | 6 |
|  |  |  | ***Mikania*** |  |  | 6 | 2 |  | 2 |  | 2 | 12 | 379 | 0 | 2 | 1 | 0 | 0 | 3 |
|  |  |  | ***Cirsium*** |  |  |  |  | 1 | 1 | 2 |  | 4 | 173 | 0 | 1 | 0 | 0 | 1 | 2 |
|  |  |  | ***Hieracium*** |  |  | 2 |  | 1 | 1 |  | 3 | 7 | 132 |  |  |  |  |  | 0 |
|  |  |  | ***Tanacetum*** |  |  | 1 | 1 |  |  |  |  | 2 | 58 |  |  |  |  |  | 0 |
|  |  |  | ***Ageratum*** |  |  |  |  | 1 |  |  |  | 1 | 20 |  |  |  |  |  | 0 |
|  |  | **Convolvulaceae** | ***Ipomoea*** |  |  |  |  | 1 |  | 2 |  | 3 | 87095 | 73 | 72 | 23 | 23 | 34 | 225 |
|  |  | **Crassulaceae** | ***Kalanchoe*** |  |  |  |  | 1 |  | 1 |  | 2 | 670 | 0 | 2 | 0 | 0 | 1 | 3 |
|  |  |  | ***Sedum*** |  |  |  |  | 1 |  |  | 1 | 2 | 145 |  |  |  |  |  | 0 |
|  |  | **Brassicaceae** | ***Brassica*** |  |  | 1 | 1 | 1 | 2 | 3 | 3 | 11 | 1047449 | 238 | 299 | 24 | 36 | 86 | 683 |
|  |  |  | ***Thlaspi*** |  |  |  |  |  | 1 |  |  | 1 | 647 |  |  |  |  |  | 0 |
|  |  |  | ***Lepidium*** |  |  | 1 |  | 3 |  | 3 | 3 | 10 | 322 |  |  |  |  |  | 0 |
|  |  |  | ***Erysimum*** |  |  | 1 |  | 1 |  |  |  | 2 | 206 |  |  |  |  |  | 0 |
|  |  |  | ***Barbarea*** |  |  |  | 1 |  |  | 2 | 7 | 10 | 31 | 0 | 6 | 0 | 0 | 0 | 6 |
|  |  |  | ***Rorippa*** |  |  | 1 |  | 1 |  | 2 | 1 | 5 | 30 |  |  |  |  |  | 0 |
|  |  |  | ***Isatis*** |  |  |  |  | 1 |  |  | 6 | 7 | 27 |  |  |  |  |  | 0 |
|  |  | **Cucurbitaceae** | ***Cucurbita*** |  |  |  |  |  |  | 1 |  | 1 | 880 | 1 | 8 | 0 | 1 | 2 | 12 |
|  |  | **Dipterocarpaceae** | ***Shorea*** | 1 |  | 102 | 34 | 3 |  | 6 | 2 | 148 | 39 | 2 | 3 | 0 | 0 | 0 | 5 |
|  |  | **Ebenaceae** | ***Diospyros*** |  |  | 14 | 14 | 40 | 5 | 19 | 4 | 96 | 14187 | 17 | 11 | 4 | 3 | 8 | 43 |
|  |  | **Ericaceae** | ***Vaccinium*** |  |  |  | 1 | 1 |  |  |  | 2 | 22401 | 110 | 22 | 4 | 8 | 6 | 150 |
|  |  |  | ***Rhododendron*** |  | 1 | 1 |  | 6 | 2 | 1 |  | 11 | 1293 | 11 | 1 | 0 | 0 | 0 | 12 |
|  |  | **Eucommiaceae** | ***Eucommia*** |  |  |  |  |  | 1 |  |  | 1 | 28456 | 84 | 28 | 1 | 5 | 17 | 135 |
|  |  | **Euphorbiaceae** | ***Euphorbia*** |  | 1 | 23 | 30 | 76 | 6 | 33 |  | 169 | 59055 | 36 | 177 | 22 | 23 | 35 | 293 |
|  |  |  | ***Jatropha*** |  |  |  | 1 | 2 |  | 1 |  | 4 | 46865 | 42 | 36 | 2 | 6 | 19 | 105 |
|  |  | **Fagaceae** | ***Quercus*** |  |  | 4 | 8 | 23 | 2 | 16 | 18 | 71 | 150124 | 207 | 163 | 24 | 21 | 73 | 488 |
|  |  |  | ***Fagus*** |  |  |  |  | 2 |  |  |  | 2 | 36740 | 50 | 47 | 9 | 4 | 28 | 138 |
|  |  |  | ***Castanopsis*** |  |  | 1 |  | 4 | 2 |  |  | 7 | 3354 | 23 | 9 | 1 | 4 | 9 | 46 |
|  |  | **Geraniaceae** | ***Geranium*** |  |  | 2 | 1 | 6 |  |  |  | 9 | 27 |  |  |  |  |  | 0 |
|  |  | **Gesneriaceae** | ***Haberlea*** |  |  |  |  |  |  | 1 |  | 1 | 373 | 0 | 0 | 0 | 0 | 2 | 2 |
|  |  |  | ***Paraboea*** |  |  | 3 | 8 | 3 |  | 4 | 5 | 23 | 2 |  |  |  |  |  | 0 |
|  |  | **Grossulariaceae** | ***Ribes*** |  |  | 2 |  | 2 |  | 1 |  | 5 | 8490 | 15 | 27 | 2 | 3 | 10 | 57 |
|  |  | **Clusiaceae** | ***Garcinia*** |  |  | 1 | 7 | 16 | 3 | 12 |  | 39 | 149 | 0 | 0 | 0 | 1 | 0 | 1 |
|  |  | **Hypericaceae** | ***Hypericum*** |  |  | 1 | 2 | 6 | 1 | 7 |  | 17 | 18 |  |  |  |  |  | 0 |
|  |  | **Illiciaceae** | ***Illicium*** |  |  |  |  | 2 |  | 2 |  | 4 | 233 | 1 | 0 | 0 | 0 | 0 | 1 |
|  |  | **Juglandaceae** | ***Juglans*** |  |  |  | 2 | 4 | 2 |  |  | 8 | 18850 | 45 | 22 | 8 | 8 | 11 | 94 |
|  |  | **Lamiaceae** | ***Salvia*** |  |  | 2 | 4 | 5 | 1 | 1 | 2 | 15 | 11766 | 12 | 11 | 3 | 4 | 5 | 35 |
|  |  |  | ***Mentha*** |  |  |  |  |  | 2 | 2 |  | 4 | 1663 | 1 | 4 | 0 | 0 | 0 | 5 |
|  |  |  | ***Lavandula*** |  |  |  |  |  |  | 1 |  | 1 | 22 |  |  |  |  |  | 0 |
|  |  | **Fabaceae** | ***Glycine*** |  |  |  |  |  |  | 2 |  | 2 | 1481192 | 143 | 208 | 21 | 32 | 79 | 483 |
|  |  |  | ***Phaseolus*** |  |  | 1 | 1 |  |  | 2 | 1 | 5 | 581936 | 58 | 85 | 20 | 11 | 42 | 216 |
|  |  |  | ***Medicago*** |  |  | 1 | 1 | 1 | 3 | 7 | 2 | 15 | 281872 | 55 | 117 | 26 | 28 | 45 | 271 |
|  |  |  | ***Arachis*** |  |  |  |  | 1 | 1 | 1 |  | 3 | 254065 | 102 | 184 | 38 | 24 | 77 | 425 |
|  |  |  | ***Lotus*** |  |  | 5 | 2 | 1 |  | 7 | 1 | 16 | 242485 | 61 | 149 | 20 | 21 | 74 | 325 |
|  |  |  | ***Vigna*** |  |  |  |  |  | 1 | 2 | 1 | 4 | 189876 | 39 | 69 | 6 | 16 | 33 | 163 |
|  |  |  | ***Trifolium*** |  |  |  |  |  | 1 | 19 |  | 20 | 53422 | 23 | 77 | 11 | 9 | 10 | 130 |
|  |  |  | ***Cicer*** |  |  |  | 2 |  |  | 2 |  | 4 | 44766 | 13 | 18 | 2 | 8 | 9 | 50 |
|  |  |  | ***Cajanus*** |  |  |  |  |  | 1 | 1 |  | 2 | 25639 | 3 | 3 | 4 | 3 | 3 | 16 |
|  |  |  | ***Acacia*** |  |  | 4 | 9 | 16 | 19 | 32 | 4 | 84 | 11569 | 14 | 11 | 1 | 0 | 5 | 31 |
|  |  |  | ***Lupinus*** |  |  |  | 1 | 1 | 4 | 14 | 5 | 25 | 9944 | 7 | 13 | 1 | 2 | 6 | 29 |
|  |  |  | ***Lathyrus*** |  |  | 1 |  |  | 1 | 8 |  | 10 | 8880 | 3 | 4 | 0 | 3 | 3 | 13 |
|  |  |  | ***Pueraria*** |  |  |  |  |  |  | 1 |  | 1 | 6365 | 7 | 10 | 1 | 6 | 5 | 29 |
|  |  |  | ***Ammopiptanthus*** |  |  | 1 |  |  |  |  |  | 1 | 5755 | 2 | 14 | 1 | 0 | 9 | 26 |
|  |  |  | ***Vicia*** |  |  | 4 | 1 |  | 1 | 9 |  | 15 | 5613 | 0 | 3 | 1 | 0 | 1 | 5 |
|  |  |  | ***Robinia*** |  |  |  |  |  |  | 3 |  | 3 | 3095 | 4 | 4 | 0 | 0 | 0 | 8 |
|  |  |  | ***Caragana*** |  |  |  |  |  |  | 3 | 1 | 4 | 1905 | 0 | 2 | 1 | 0 | 1 | 4 |
|  |  |  | ***Prosopis*** |  |  |  |  | 1 | 2 | 2 | 3 | 8 | 1599 | 3 | 0 | 0 | 1 | 1 | 5 |
|  |  |  | ***Oxytropis*** |  |  |  |  |  |  | 6 | 2 | 8 | 1245 | 0 | 1 | 0 | 0 | 0 | 1 |
|  |  |  | ***Macrotyloma*** |  |  |  |  |  |  | 1 |  | 1 | 1025 | 0 | 0 | 0 | 1 | 0 | 1 |
|  |  |  | ***Copaifera*** |  |  |  |  | 3 |  | 1 |  | 4 | 716 | 2 | 2 | 0 | 0 | 1 | 5 |
|  |  |  | ***Sesbania*** |  |  |  |  | 1 |  | 8 | 1 | 10 | 374 | 0 | 1 | 0 | 0 | 1 | 2 |
|  |  |  | ***Stylosanthes*** |  |  |  |  |  |  | 1 |  | 1 | 350 | 0 | 0 | 0 | 0 | 0 | 0 |
|  |  |  | ***Hymenaea*** |  |  |  |  | 1 |  | 3 |  | 4 | 299 | 0 | 1 | 0 | 0 | 0 | 1 |
|  |  |  | ***Leucaena*** |  |  |  | 3 | 4 | 1 |  |  | 8 | 150 | 0 | 0 | 1 | 0 | 0 | 1 |
|  |  |  | ***Astragalus*** | 1 |  | 6 | 7 | 4 | 4 | 54 |  | 76 | 125 |  |  |  |  |  | 0 |
|  |  |  | ***Hedysarum*** |  |  |  |  |  | 1 | 3 | 1 | 5 | 6 |  |  |  |  |  | 0 |
|  |  | **Linaceae** | ***Linum*** |  |  | 1 |  | 2 |  |  | 1 | 4 | 286856 | 33 | 128 | 20 | 6 | 8 | 195 |
|  |  | **Lythraceae** | ***Punica*** |  |  |  |  | 1 |  | 1 |  | 2 | 2383 | 6 | 3 | 1 | 0 | 3 | 13 |
|  |  |  | ***Sonneratia*** |  |  | 2 |  |  | 1 | 4 |  | 7 | 249 | 2 | 0 | 0 | 0 | 0 | 2 |
|  |  | **Malvaceae** | ***Hibiscus*** |  |  | 2 | 2 | 2 | 1 | 3 | 2 | 12 | 47 |  |  |  |  |  | 0 |
|  |  |  | ***Abutilon*** |  |  | 3 |  | 1 |  |  |  | 4 | 5 |  |  |  |  |  | 0 |
|  |  | **Moraceae** | ***Ficus*** |  |  | 3 | 10 | 11 | 1 | 23 |  | 48 | 9296 | 21 | 37 | 0 | 5 | 17 | 80 |
|  |  |  | ***Artocarpus*** |  |  |  |  | 5 |  |  |  | 5 | 3 |  |  |  |  |  | 0 |
|  |  | **Myrtaceae** | ***Eucalyptus*** |  |  | 1 | 1 |  |  |  |  | 2 | 167436 | 228 | 197 | 24 | 19 | 65 | 533 |
|  |  |  | ***Myrciaria*** |  |  |  |  | 3 |  |  |  | 3 | 8 |  |  |  |  |  | 0 |
|  |  | **Oleaceae** | ***Fraxinus*** |  |  | 1 |  |  | 1 |  |  | 2 | 12083 | 9 | 2 | 1 | 1 | 4 | 17 |
|  |  |  | ***Olea*** |  |  |  |  | 1 |  |  | 1 | 2 | 9959 | 0 | 2 | 0 | 1 | 4 | 7 |
|  |  | **Paeoniaceae** | ***Paeonia*** |  |  |  | 1 |  |  |  |  | 1 | 2204 | 2 | 2 | 0 | 0 | 1 | 5 |
|  |  | **Papaveraceae** | ***Papaver*** |  |  |  |  | 1 |  |  |  | 1 | 26523 | 21 | 28 | 4 | 5 | 6 | 64 |
|  |  | **Plantaginaceae** | ***Plantago*** |  |  | 2 | 2 | 1 |  | 1 | 1 | 7 | 3 |  |  |  |  |  | 0 |
|  |  | **Platanaceae** | ***Platanus*** |  |  |  |  | 1 |  | 1 |  | 2 | 7 |  |  |  |  |  | 0 |
|  |  | **Plumbaginaceae** | ***Limonium*** |  |  | 5 | 5 | 4 | 5 | 3 | 1 | 23 | 5534 | 0 | 2 | 1 | 1 | 0 | 4 |
|  |  |  | ***Plumbago*** |  |  |  |  |  |  | 1 |  | 1 | 1842 | 2 | 1 | 0 | 0 | 6 | 9 |
|  |  | **Podostemaceae** | ***Polypleurum*** |  |  |  |  | 1 | 3 | 6 | 4 | 14 | 9679 | 0 | 0 | 1 | 0 | 0 | 1 |
|  |  | **Polygonaceae** | ***Persicaria*** |  |  |  |  |  |  | 10 |  | 10 | 3538 | 5 | 4 | 2 | 0 | 2 | 13 |
|  |  | **Primulaceae** | ***Primula*** |  |  |  | 1 | 1 | 1 | 3 |  | 6 | 5672 | 6 | 4 | 0 | 3 | 5 | 18 |
|  |  |  | ***Aegiceras*** |  |  |  |  |  | 1 | 1 |  | 2 | 68 |  |  |  |  |  | 0 |
|  |  | **Ranunculaceae** | ***Aquilegia*** |  |  | 2 |  |  |  | 2 | 1 | 5 | 115666 | 73 | 217 | 35 | 14 | 46 | 385 |
|  |  |  | ***Adonis*** |  |  |  |  |  |  |  | 1 | 1 | 4189 | 0 | 2 | 0 | 0 | 2 | 4 |
|  |  | **Rhamnaceae** | ***Ziziphus*** |  |  |  | 1 | 3 |  | 1 | 1 | 6 | 3401 | 5 | 7 | 0 | 0 | 0 | 12 |
|  |  | **Rhizophoraceae** | ***Bruguiera*** |  |  | 1 |  |  |  | 5 |  | 6 | 22543 | 48 | 49 | 6 | 8 | 7 | 118 |
|  |  |  | ***Rhizophora*** |  |  |  |  |  | 1 | 5 |  | 6 | 331 |  |  |  |  |  | 0 |
|  |  |  | ***Ceriops*** |  |  |  |  |  | 1 | 3 |  | 4 | 101 |  |  |  |  |  | 0 |
|  |  |  | ***Kandelia*** |  |  |  |  |  |  | 2 |  | 2 | 1 |  |  |  |  |  | 0 |
|  |  | **Rosaceae** | ***Malus*** |  |  |  | 1 | 2 |  |  | 4 | 7 | 336190 | 183 | 67 | 17 | 24 | 31 | 322 |
|  |  |  | ***Prunus*** |  |  | 2 | 7 | 7 | 3 | 7 | 4 | 30 | 111788 | 217 | 63 | 17 | 15 | 32 | 344 |
|  |  |  | ***Rubus*** |  |  |  |  | 2 |  |  |  | 2 | 3061 | 9 | 10 | 1 | 0 | 6 | 26 |
|  |  |  | ***Pyrus*** |  |  | 3 | 1 | 1 | 3 |  | 5 | 13 | 1942 | 11 | 2 | 1 | 0 | 1 | 15 |
|  |  |  | ***Photinia*** |  |  |  |  | 2 |  |  |  | 2 | 44 |  |  |  |  |  | 0 |
|  |  |  | ***Potentilla*** |  |  |  |  | 1 | 1 |  | 2 | 4 | 1 |  |  |  |  |  | 0 |
|  |  | **Rubiaceae** | ***Coffea*** |  |  |  | 2 | 8 |  |  |  | 10 | 254474 | 102 | 94 | 19 | 16 | 27 | 258 |
|  |  |  | ***Oldenlandia*** | 1 |  |  |  | 1 |  | 4 | 2 | 8 | 1117 | 0 | 1 | 1 | 0 | 0 | 2 |
|  |  |  | ***Psychotria*** |  |  | 13 | 17 | 29 | 4 | 2 | 10 | 75 | 10 |  |  |  |  |  | 0 |
|  |  | **Rutaceae** | ***Citrus*** |  |  | 1 |  |  |  |  |  | 1 | 568700 | 126 | 123 | 26 | 21 | 46 | 342 |
|  |  | **Salicaceae** | ***Populus*** |  |  | 1 | 1 | 1 | 1 | 1 |  | 5 | 422517 | 176 | 207 | 51 | 45 | 88 | 567 |
|  |  |  | ***Salix*** |  |  | 1 | 1 | 2 | 1 | 3 |  | 8 | 707 |  |  |  |  |  | 0 |
|  |  | **Santalaceae** | ***Santalum*** | 1 |  |  | 1 | 2 |  |  |  | 4 | 95 |  |  |  |  |  | 0 |
|  |  | **Sapindaceae** | ***Paullinia*** |  |  |  |  | 1 |  |  |  | 1 | 14888 | 5 | 3 | 0 | 1 | 2 | 11 |
|  |  |  | ***Dimocarpus*** |  |  |  |  |  | 1 |  |  | 1 | 66 | 1 | 0 | 0 | 0 | 0 | 1 |
|  |  | **Scrophulariaceae** | ***Antirrhinum*** |  |  | 1 | 1 |  |  |  |  | 2 | 25310 | 11 | 30 | 5 | 7 | 12 | 65 |
|  |  |  | ***Mimulus*** |  |  |  |  |  |  | 2 |  | 2 | 279620 | 225 | 253 | 37 | 23 | 52 | 590 |
|  |  |  | ***Torenia*** |  |  |  |  |  |  | 1 |  | 1 | 256 | 0 | 0 | 1 | 2 | 0 | 3 |
|  |  | **Solanaceae** | ***Solanum*** |  |  | 6 | 6 | 11 | 14 | 2 | 8 | 47 | 725742 | 71 | 126 | 17 | 25 | 85 | 324 |
|  |  |  | ***Nicotiana*** |  |  |  |  |  |  | 1 |  | 1 | 413102 | 111 | 148 | 16 | 25 | 65 | 365 |
|  |  |  | ***Withania*** |  |  |  |  |  |  | 2 |  | 2 | 741 | 0 | 0 | 0 | 1 | 0 | 1 |
|  |  | **Tamaricaceae** | ***Tamarix*** |  |  |  |  |  |  | 1 |  | 1 | 22712 | 8 | 6 | 5 | 4 | 4 | 27 |
|  |  | **Theaceae** | ***Camellia*** |  |  |  | 1 | 10 |  |  |  | 11 | 49902 | 109 | 43 | 9 | 11 | 26 | 198 |
|  |  | **Tiliaceae** | ***Corchorus*** |  |  |  |  |  |  | 1 |  | 1 | 840 | 0 | 1 | 0 | 0 | 0 | 1 |
|  |  | **Tropaeolaceae** | ***Tropaeolum*** |  |  | 1 | 1 | 6 |  |  | 1 | 9 | 10507 | 0 | 3 | 0 | 0 | 1 | 4 |
|  |  | **Ulmaceae** | ***Ulmus*** |  |  | 1 | 1 | 2 |  |  |  | 4 | 1277 | 0 | 1 | 0 | 0 | 0 | 1 |
|  |  | **Apiaceae** | ***Daucus*** |  |  |  |  |  |  |  | 1 | 1 | 18137 | 3 | 1 | 0 | 0 | 3 | 7 |
|  |  |  | ***Centella*** |  |  |  |  |  |  | 1 |  | 1 | 443 | 2 | 3 | 0 | 0 | 1 | 6 |
|  |  |  | ***Apium*** |  |  | 1 |  |  | 1 | 3 |  | 5 | 2336 | 1 | 3 | 0 | 0 | 0 | 4 |
|  |  |  | ***Bupleurum*** |  |  | 3 | 1 | 1 |  |  |  | 5 | 237 | 5 | 2 | 0 | 0 | 0 | 7 |
|  |  |  | ***Angelica*** |  |  |  |  |  |  | 1 |  | 1 | 63 |  |  |  |  |  | 0 |
|  |  | **Urticaceae** | ***Boehmeria*** |  |  |  |  |  | 1 |  |  | 1 | 421 | 0 | 1 | 0 | 0 | 1 | 2 |
|  |  | **Violaceae** | ***Viola*** | 1 |  | 2 | 1 | 1 | 1 | 1 |  | 7 | 49 |  |  |  |  |  | 0 |
|  |  | **Vitaceae** | ***Vitis*** |  |  |  |  |  |  | 1 |  | 1 | 479626 | 69 | 73 | 13 | 23 | 47 | 225 |

**Table S1:** List of the IUCN plant genera mined for EST-SSRs with raw results. Number of species for each genera within each IUCN category (EX = extinct, EW = extinct in the wild, CR =critically endangered, EN = endangered, VU = vulnerable, NT =near threatened, LC =least concern, DD =data deficient). N EST, number of EST sequences downloaded for each genera; di-, tri-, tetra-, penta- and hexa- denotes type of SSRs and total indicates the total number of SSRs with primer information found for each genera.
