# Supplementary material for: Mining microsatellite markers from public expressed sequence tags databases for the study of threatened plants
Source: BMC Genomics. 2015 Oct 13;16:781. doi: 10.1186/s12864-015-2031-1 (PMC4603344; doi:10.1186/s12864-015-2031-1)
Supplement: Additional file 3: Table S2. — Impact of the mining criteria (Type I or relaxed parameters) in the total number of EST-SSRs detected in ten randomly selected genera. (DOCX 13 kb) [file 12864_2015_2031_MOESM3_ESM.docx]

**Table S2**: Impact of the mining criteria (Type I or relaxed parameters) in the total number of EST-SSRs detected in ten randomly selected genera.

|  | **Type I SSR** | | | | | | **Relaxed parameters** | | | | | |
| --- | --- | --- | --- | --- | --- | --- | --- | --- | --- | --- | --- | --- |
| **Genus** | **dinucleotides** | **trinucleotides** | **tetranucleotides** | **pentanucleotides** | **hexanucleotides** | **total** | **dinucleotides** | **trinucleotides** | **tetranucleotides** | **pentanucleotides** | **hexanucleotides** | **total** |
| **Chamaecyparis^1^** | 0 | 2 | 1 | 1 | 2 | 6 | 5 | 4 | 1 | 1 | 2 | 13 |
| **Pinus^1^** | 38 | 29 | 6 | 15 | 64 | 157 | 771 | 53 | 10 | 15 | 64 | 219 |
| **Gingko^1^** | 16 | 7 | 1 | 1 | 6 | 31 | 25 | 10 | 1 | 1 | 6 | 43 |
| **Setaria^3^** | 7 | 17 | 4 | 11 | 3 | 46 | 21 | 21 | 6 | 11 | 3 | 62 |
| **Asparagus^3^** | 30 | 32 | 1 | 2 | 4 | 43 | 16 | 20 | 1 | 2 | 4 | 69 |
| **Cicer^4^** | 13 | 18 | 2 | 8 | 9 | 50 | 28 | 30 | 4 | 8 | 9 | 79 |
| **Primula^4^** | 6 | 4 | 0 | 3 | 5 | 18 | 19 | 6 | 0 | 3 | 5 | 33 |
| **Vaccinium^4^** | 110 | 22 | 4 | 8 | 6 | 150 | 211 | 8 | 4 | 8 | 6 | 311 |
| **Actinidia^4^** | 278 | 54 | 18 | 13 | 55 | 410 | 543 | 128 | 18 | 13 | 55 | 757 |

The relaxed parameters consisted in at least four repetition for each type of SSR as recommended in the default parameters of QDD. Numbers in superscript indicates the taxonomic group of the genus ^1^ for Acrogymnospermae, ^2^ for Magnoliidae, ^3^ for Monocotyledoneae, ^4^ for Eudicotyledoneae
