# Supplementary material for: Mining microsatellite markers from public expressed sequence tags databases for the study of threatened plants
Source: BMC Genomics. 2015 Oct 13;16:781. doi: 10.1186/s12864-015-2031-1 (PMC4603344; doi:10.1186/s12864-015-2031-1)

**Instructions for the database use.**

Files included in the Dryad repository: Instructions file, Table S1 and 194 excel files (one for each genus included in the .rar file Type I EST_SSR).

a) Instructions file: this file contains a brief explanation for the users.

b) Table S1: table including all the genera (257) that were mined for EST-SSR. In this file the user can find information about how many species of the genus are included in the IUCN list and in which category. Moreover, the number of EST-SSRs of each type found, and the total number of EST-SSR, are also detailed.

c) Excel files: each file contains information for one genus. The name of the genus corresponds with the name of the file. In each file the user can find detailed information about the EST-SSRs and the primers. Each column has a headline that identifies the type of information within that column. The different columns within the excel file are explained in order below:

- Seq_code: ID of the sequence. Sequence code followed by a number to distinguish different target microsatellites of the same sequence. When starting with gi that belongs to the original ID from the GenBank. By imputing the number in the EST Database from NCBI the user can check the original record. E.g. ID gi40839849_0 belongs to AMF123_E04 Acacia mangium flower subtracted cDNA library Acacia mangium cDNA, mRNA sequence. Please note that before searching for the number the gi and the _0 has to be deleted, only the number between this corresponds with the GenBank ID. When the ID is labelled as consXXX or concatXXX corresponds with sequences with redundancy, in this cases the original ID from GenBank were deleted by the software.

- Min_PCR_product_size: The minimum length of the PCR product set for Primer3.

- PCR_product_size: Size of the expected PCR product.

- Primer_pair_penalty: The value of the penalty for this pair of primers (lower is better).

- Primer_left_penalty: The penalty value of the left primer.

- Primer_rigth_penalty: The penalty value of the left primer.

- Primer_left_sequence: Nucleotide sequence from primer left.

- Primer_rigth_sequence: Nucleotide sequence from primer right.

- Primer_left_pos: Starting position of primer left in the targeted sequence (bp).

- Primer_left_length: Length of the primer left in bp.

- Primer_rigth_pos: Starting position of primer right in the targeted sequence (bp).

- Primer_rigth_length: Length of the primer right in bp.

- Primer_rigth_TM: The melting TM for the primer right.

- Primer-left_TM: The melting TM for the primer left.

- Primer_left_GC_percent: The percent GC for the left primer.

- Primer_right_GC_percent: The percent GC for the right primer.

- Primer_left_self_any: The calculated value for the tendency of the primer left to bind to itself (interfering with target sequence binding).

- Primer_rigth_self_any: The calculated value for the tendency of the primer right to bind to itself (interfering with target sequence binding).

- Primer_left_self_end: The calculated value for the tendency of the 3'-END to bind to an identical primer. This is critical for primer quality because it allows primers use itself as a target and amplify a short piece (forming a primer-dimer). These primer are then unable to bind and amplify the target sequence.

- Primer_rigth_self_end: The calculated value for the tendency of the 3'-END to bind to an identical primer. This is critical for primer quality because it allows primers use itself as a target and amplify a short piece (forming a primer-dimer). These primer are then unable to bind and amplify the target sequence.

- Primer_left_end_stability: The value is the delta G of disruption of the five 3' bases of the primer.

- Primer_rigth_end_stability: The value is the delta G of disruption of the five 3' bases of the primer.

- Primer_pair_compl_any: The calculated value for the tendency of the primer pair to bind to each other (interfering with target sequence binding). The inter-pair complementarity measures over the complete primer for selected left and right primer.

- Primer_pair_compl_end: The calculated value for the tendency of the 3'-ENDs of a primer pair to bind to each other. This is critical for primer quality because it allows primers use itself as a target and amplify a short piece (forming a primer-dimer). These primer are then unable to bind and amplify the target sequence. The inter-pair complementarity measures for the ends of selected left and right primer.

- First_pos: Baise pair where the SSR starts in the targeted sequence.

- Length_BP: Length in bp of the SSR.

- Length_rep: Length of the motif repeated.

- Motif: SSR motif.

- Trans_motif: Reverse motif of the SSR.

- AD column: This column has no title. The option identified with the label “*best*” is supposed to be the EST-SSR with the primer pair with less penalty. In the image below we can see that for the sequence with the ID gi40839849_0 there are 11 primer options for the same EST-SSR but only the one in line 274 has the label “*best”*. With the option sorting in the excel file, one can select only the “*best*” options. The total number of EST-SSR found for a genus correspond with the number of “*best*” primer pairs.


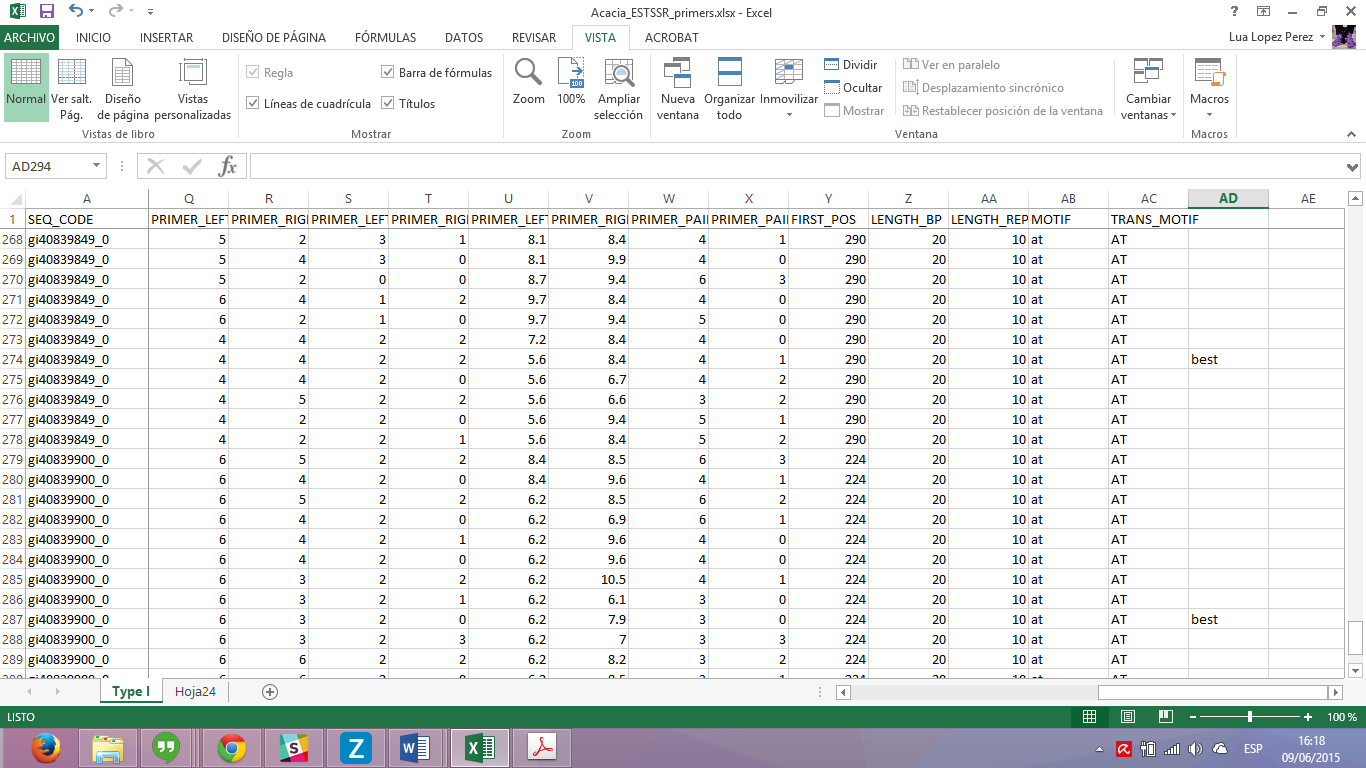

Supplement: Additional file 4: — Instructions for the database use. (DOCX 147 kb) [file 12864_2015_2031_MOESM4_ESM.docx]
